# Supplementary material for: A Model of Compound Heterozygous, Loss-of-Function Alleles Is Broadly Consistent with Observations from Complex-Disease GWAS Datasets
Source: PLoS Genet. 2017 Jan 19;13(1):e1006573. doi: 10.1371/journal.pgen.1006573 (PMC5289629; doi:10.1371/journal.pgen.1006573)
Supplement: S1 Text — (PDF) [file pgen.1006573.s001.pdf]

S1 Text

Population genetic modeling of complex traits

Forward-in-time simulations, as in [20, 22, 25, 74] and [S1–S4] , explicitly model the allele frequencies and effect sizes of mutations in a genomic region with neutral sites, selected sites and recombination. Within the forward simulation framework, methods differ in their approach to assigning phenotypes to particular genotypes. One approach, based on the work of [18], models a mutation's effect on fitness as a pleiotropic consequence of its effect on trait values. In that model, by specifying a parameter τ, the user establishes the shape of relationship between the fitness effects and expected trait effects of variants [20, 22] and [S4, S6]. There is another term, ϵ, which adds noise to the fitness-trait relationship; together τ and ϵ determine the correlation between fitness effects and trait effects. A related approach that builds off of much earlier work by [S7], models trait and fitness effects as coming from a bivariate gamma distribution with a specified correlation parameter ρ [25]. In both approaches the disease-trait itself is not a component of fitness and thus standing variation may be dominated by occasional large trait effect mutations with small fitness effects that can reach intermediate frequency. The extent to which this occurs is dependent on the degree of correlation between fitness and trait effects. Furthermore, both approaches indicate that an intermediate degree of correlation between complex disease traits and fitness is most plausible [20, 25, 65](although [65] is not a population genetic simulation based implementation of [18], a similar conclusion was reached.)

The approach in [36] is similar to typical models [S10–S14] and [71] of selection on quantitative traits where phenotype is the sum of genetic and environmental components and is subjected to Gaussian stabilizing selection. A key difference between [36] and the typical quantitative trait models is that all causal mutations are unconditionally deleterious and the gene action model exhibits gene-based recessivity, i.e, allelic non-complementation. While the work in [36] presented a new genetic model, it was limited because only that model was explored under a single demographic scenario (constant sized population). To our knowledge, there has not been a joint analysis of the effect of genetic model and demography on the predicted outcomes of GWAS. Thus, we extend the approach of [36] by including a model of recent population expansion and a set of genetic models.

Heritability and genetic load under population growth

Before deeply exploring the predicted genetic architecture of a trait under each model, we looked at two key mean values: total genetic variance and load. The genetic variance underlying a trait is, in part, determined by the outcome of mutation-selection balance. Approximations for the expected genetic variance under models of stabilizing selection with Gaussian mutational effect sizes and additive gene action have been derived previously [S15] and [71].

According to the house-of-cards(HOC) approximation, when the variance in mutational effect sizes is large compared to total genetic variance, the genetic variance will be dependent only on the mutation rate, μ, and the intensity of stabilizing selection, $1/\sigma_s^2$. Here, μ refers to the total mutation rate in the "gene region" (per gamete, per generation), with mutations arising according to an infinitely-many sites scheme [88]. In a diploid species, $V_G \approx 4\mu\sigma_s^2$ for an additive trait, and $V_G \approx 2\mu\sigma_s^2$ for a recessive trait [71, 89]. By keeping σ_s^2 and μ constant, we can modulate the broad sense heritability ($H^2 = (V_G)/(V_G + V_E)$) by changing the environmental variance, $V_E = \sigma_e^2$. These approximations are expected to hold for arbitrary probability distributions of mutational effect sizes [S17]; however all distributions discussed in [S17] are reflected

about zero. Here, as in [36], we draw the effect sizes of causal mutation from a standard exponential distribution, modeling unconditionally deleterious mutations. As previously shown in [36], heritability approaches the value expected under the HOC approximation when the variance in effect sizes (λ^2) is large (S1 Fig).

Previous work, under additive genetic models, on the impact of population growth on the genetic architecture of complex traits suggests that mean heritability is constant under growth [21, 22]. We confirm this in S1 Fig, showing that mean broad-sense heritability, $H^2 = (V_G)/(V_P)$, initially increases as λ, the mean effect of a new deleterious mutation, increases and then approximately reaches the same level as models with constant population size. This general trend is observed under each genetic model, but the MR model is qualitatively different in its behavior under population growth. The MR model predicts a broad sense heritability under growth of about 90% of constant sized population levels when $\lambda = 0.01$, and 50% when $\lambda = 0.5$ (S2 Fig).

The degree to which recent demographic history has impacted the distribution of genetic variance over risk allele frequency in human populations is still unclear. One line of evidence may come from the study of genetic load in humans. If fitness effects and trait effects of variants are correlated, then the composition of the genetic load of deleterious mutations in the population is highly relevant to the genetic architecture of that trait. Comparisons between populations with different demographic histories provide insight into the impact of demography on genetic load. The influential study of [S20] found there to be both more non-synonymous relative to synonymous variants and a higher average number of homozygous non-synonymous sites in European Americans than African Americans. Later studies showed empirically and through simulations that the mean allele frequency of deleterious mutations is not impacted by recent demographic history [21, S21, S22]. Simulations presented by [S23] suggest that load is expected to increase during a range expansion, without an increase in mean frequency of deleterious alleles, due to an increase in homozygosity at deleterious recessive sites. By invoking expansion load theory and empirical data from multiple human populations, [S24] argue that load is increased in non-African populations due to serial bottlenecks during range expansion after the out of Africa event. While arguments about genetic load are sensitive to choice of metric [90], and the empirical evidence supporting one view or another is still lacking, it does appear that any differences between current human populations due to past population bottlenecks is likely to be small.

Our results show that genetic burden (load), as measured by the average relative deviation from optimum fitness, of the population is also unaffected by recent population history under the AC model (S3 Fig), as shown in [21, 22, 91]. We find this same behavior under the GBR model, but not under the MR model. Under rapid population expansion, the load decreases slightly (at most 2%). As λ increases the load increases under the additive model in both demographic scenarios. Load is effectively constant over the range of λ under the GBR model. Increasing the heritability of the trait decreased the magnitude of the genetic load, but had no interaction with the effects of demography or increasing mean effect size.

We also find that the dynamics of load under more complex demographic models involving multiple bottlenecks and recent growths behaves as expected from previous literature [S25] and [21, 91]. The Tennessen [40] demographic model is characterized by an ancient expansion, two recent bottlenecks and subsequent rapid population expansion. In models with strong recessive selection the load should increase immediately after the bottleneck, then decay due to the purging of deleterious alleles in homozygotes [S25]. Upon population rexpansion, in models with strong recessive selection, we expect the load to further drop below equilibrium levels [21, 91]. We observe this pattern most clearly in the multiplicative recessive models (cMR or

iMR($h = 0.1$)) when λ is large S18 Fig. In S19 Fig we also show the Burden Ratio (B_r) [91] calculated relative to a model with no bottleneck or growth, which provides a clear visualization of the aformentioned dynamics. Further, in agreement with Simons et al [21], the number of deleterious alleles per individual decreases following the bottleneck under strong recessive selection. This results in an increase in the B_r calculated using the number of alleles S20 Fig following the bottleneck. We note that because B_r is calculated by comparing two sets of simulations, it may not be exactly equal to one when comparing time points at which the two simulations share identical demographic histories. This is especially the case for the B_r calculated using fixed load which, being small, has high relative variance.

From these results we can conclude that the AC and GBR models are fairly comparable with respect to total genetic variance and genetic load. Therefore, the remaining differences, which we highlight in the main text, between the AC and GBR model can be attributed to the fine scale composition of the genetic variance in the population. In other words, the AC and GBR model differ in how the genetic variance and load are accounted for, despite the total amounts being roughly equivalent. However, the MR model is qualitatively different in its behavior under population growth. This makes comparison to the MR model somewhat difficult. However, there are important reasons to explore it further. Based on first principles, the application of the MR model in simulation of a single gene region is inappropriate. However, it would be appropriate for simulating each mutation as a variant of a distinct functional genomic unit. It is also the most analytically tractable model of recessivity in population genetics, and as such it is our best reference point for comparison to the GBR model.

The approximate distribution of fitness effects

Here, fitness is a function of phenotype and so the distribution of fitness effects of newly arising mutations is dependent on the state of the population. However, we can achieve an approximate result by assuming that large effect mutations are rare and considering the effect of a new mutation on an unaffected genetic background [S26]. This approximation is likely to be most accurate for large values of λ. In this case, we can find the exact distribution of fitness effects given the distribution of trait effects by a simple change of variables. We will assume a fitness model where fitness is 1, $1 - sh$ and $1 - s$ for 0,1, or 2 copies of the deleterious mutation. Although, excepting complete recessive selection, the expected allele frequency trajectories are determined by sh we focus on the distribution of s and emphasize that selection is still recessive ($h < 0.5$) under the additive phenotypic model(S15 Fig).

Let $f_z(z)$ describe the density of mutant phenotypic effects and $s(z)$ describe the fitness of a homozygote for a deleterious allele. We can find the density ($f_s(s)$) and cumulative distribution ($F_s(s)$) of s by change of variables.

$$f_z(z) \sim Exp(\frac{1}{\lambda})$$

$$s(z) = 1 - e^{-\frac{(2*z)^2}{2}}$$

$$f_s(s) = f_z(s^{-1}(s))\frac{d}{ds}s^{-1}(s)$$

$$s^{-1}(s) = \frac{\sqrt{-2\log(1-s)}}{2}$$

$$f_s(s) = \frac{\frac{e^{-\frac{\sqrt{-\log(1-s)}}{\sqrt{2}\lambda}}}{\lambda}}{2\sqrt{2}(1-s)\sqrt{-\log(1-s)}}$$

$$F_s(s) = 1 - e^{\frac{\sqrt{-log(1-s)}}{\sqrt{(2)}\lambda}}$$

We checked this result via simple sampling in R [96] (S22 Fig), using the population size scaled parameter $2Ns$. Across the range of λ simulated the distribution of fitness effects spans multiple selective regimes. In all cases there will be some weakly and strongly selected mutations. When $\lambda < 0.1$ there will also be a considerable proportion of nearly neutral mutations. Again, we emphasize that the degree of recessivity can have an important effect here, as the distribution of $2Nsh$ will be shifted to the left in S22 Fig. It is also important to observe the appearance of a mass of lethal mutations $s \approx 1$ as λ gets larger in S23 Fig. These approximate distributions of fitness effects reveal the relative impact of mutations in different selective regimes in each model. In general, the simulated frequency spectra (see S7 Fig, S8 Fig, and S9 Fig) and genetic loads (see S3 Fig and S18 Fig) are in agreement with the expecations under the approximate distribution of fitness effects.

Choice of genetic model effects key population genetic signatures

In this section, we explore how the choice of genetic model impacts the site frequency spectrum for risk variants. Mean genetic load decreases with degree of dominance (S3 Fig). Recessive deleterious mutations segregate to higher frequencies in the population without increasing genetic load (S5 Fig). The MR model demonstrates a slight decrease in load under growth (S3 Fig). Similarly, the additive model has greater skew and kurtosis for both the number of mutations and the genetic value of a gamete over the range of λ and demographic models (S11 Fig and S12 Fig).The increase skew and kurtosis implies that total genetic load in the additive models is dominated by rare large excursions from the population mean.

Population expansion has been shown to impact the site frequency spectrum [54, 55, 59, 99]. In general, we expect to find an increase in rare private mutations under a rapid population expansion scenario. We find all of our models showcase the expected pattern (S7 Fig), but there are consistent and important differences between models. In S7 Fig, the site frequency spectrum of risk variants from a population sample (n=100) shows a dependency on population growth, mean effect size λ, and genetic model. Population growth increases the proportion of singletons for all genetic models and values of λ. Increasing the value λ increases the proportion of singletons in each genetic model and demographic scenario, but the increase is qualitatively dependent on genetic model and independent of demography. The recessive models show the strongest dependence on λ. When λ is small the recessive models show fewer singletons as compared to the additive model, but as λ increases the relative proportion of singletons between recessive and additive genetic model increases. When the value of λ is large, the recessive models show more singletons than the additive model. The GBR model shows more singletons than the MR model in all cases. S8 Fig shows the site frequency spectrum for non-risk variants, which demonstrates a dependence on population growth, shifting towards low-frequency sites, but shows no dependence on genetic model or λ. Since the neutral variant site frequency spectrum is

consistent across models we determine that the difference in linked selection between models is not important in the relevant recombination rate regime.

Regression based estimates of genetic variance

For Fig 1 and S4 Fig, we performed linear regression of the genetic component of phenotype onto genotypes. This provided an estimate of distribution of genetic variance over risk allele frequency. Under an additive model and Hardy-Weinberg linkage equilibrium (HWLE), these estimates are identical to the classic result $V_G = 2pq\alpha^2$ [9]. To demonstrate this we simulated genotype data at 1000 independent markers for 5000 individuals in R [96]. Population frequencies were drawn from the constant population size neutral allele frequency distribution. Allele counts for individuals were binomial samples of size two with probability of success equal to the population frequency of the minor allele. Effects sizes were sampled randomly from an exponential distribution with mean $\lambda = 0.1$, to mimic our simulations in the main text. Regressions were performed only on markers which were not fixed in the sample. S14 Fig shows the regression estimate as a function of its expected value $2pq\alpha^2$. There is some noise for markers with low total variance explained, which is due to random deviations from HWLE.

Supporting Information References

[S1] Price AL, Kryukov GV, de Bakker PIW, Purcell SM, Staples J, Wei LJ, et al. Pooled association tests for rare variants in exon-resequencing studies. American journal of human genetics. 2010 jun;86(6):832–8. Available from: http://www.cell.com/article/S0002929710002077/fulltext.

[S2] King CR, Rathouz PJ, Nicolae DL. An evolutionary framework for association testing in resequencing studies. PLoS genetics. 2010 nov;6(11):e1001202. Available from: http://journals.plos.org/plosgenetics/article?id=10.1371/journal.pgen.1001202.

[S3] Browning SR, Thompson EA. Detecting rare variant associations by identity-by-descent mapping in case-control studies. Genetics. 2012 apr;190(4):1521–31. Available from: http://www.genetics.org/content/early/2012/01/18/genetics.111.136937.

[S4] North TL, Beaumont MA. Complex trait architecture: the pleiotropic model revisited. Scientific reports. 2015 jan;5:9351. Available from: http://www.nature.com/srep/2015/150320/srep09351/full/srep09351.html.

[S5] Eyre-Walker A. Evolution in health and medicine Sackler colloquium: Genetic architecture of a complex trait and its implications for fitness and genome-wide association studies. Proceedings of the National Academy of Sciences of the United States of America. 2010;107 Suppl:1752–1756.

[S6] Moutsianas L, Agarwala V, Fuchsberger C, Flannick J, Rivas MA, Gaulton KJ, et al. The power of gene-based rare variant methods to detect disease-associated variation and test hypotheses about complex disease. PLoS genetics. 2015 apr;11(4):e1005165. Available from: http://journals.plos.org/plosgenetics/article?id=10.1371/journal.pgen.1005165.

[S7] Keightley PD, Hill WG. Variation Maintained in Quantitative Traits with Mutation-Selection Balance: Pleiotropic Side-Effects on Fitness Traits. Proceedings of the Royal Society B: Biological Sciences. 1990

nov;242(1304):95–100. Available from: http://rspb.royalsocietypublishing.org/content/242/1304/95.

[S8] Caballero A, Tenesa A, Keightley PD. The Nature of Genetic Variation for Complex Traits Revealed by GWAS and Regional Heritability Mapping Analyses. Genetics. 2015 oct;201(4):1601–13. Available from: http://genetics.org/content/201/4/1601.abstract.

[S9] Thornton KR, Foran AJ, Long AD. Properties and Modeling of GWAS when Complex Disease Risk Is Due to Non-Complementing, Deleterious Mutations in Genes of Large Effect. PLoS Genetics. 2013;9(2).

[S10] Haldane JBS. The measurement of natural selection. Proc 9th Int Congr Genet. 1954;1:480–487.

[S11] Latter BDH. Natural selection for an intermediate optimum. Australian Journal of Biological Sciences. 1960;13:30–35. Available from: http://www.cabdirect.org/abstracts/19600101674.html;jsessionid=3B1562EB69031455E4C7BB05F3D8B699?freeview=true.

[S12] Crow JF, Kimura M. The theory of genetic loads. Proc XI Int Congr Genet. 1964;p. 495–505.

[S13] Kingman JFC. A Simple Model for the Balance between Selection and Mutation. Journal of Applied Probability. 1978 mar;15(1):1–12. Available from: http://www.jstor.org/stable/3213231.

[S14] Fleming WH. Equilibrium Distributions of Continuous Polygenic Traits. SIAM Journal on Applied Mathematics. 1979 jul;Available from: http://epubs.siam.org/doi/abs/10.1137/0136014.

[S15] Kimura M. A stochastic model concerning the maintenance of genetic variability in quantitative characters. Proceedings of the National Academy of Sciences of the United States of America. 1965 sep;54(3):731–6. Available from: http://www.pubmedcentral.nih.gov/articlerender.fcgi?artid=219735{&}tool=pmcentrez{&}rendertype=abstract.

[S16] Kimura M. The number of heterozygous nucleotide sites maintained in a finite population due to steady flux of mutations. Genetics. 1969;61(694):893–903.

[S17] Burger R, Hofbauer J. Mutation load and mutation-selection-balance in quantitative genetic traits. Journal of Mathematical Biology. 1994 feb;32(3):193–218. Available from: http://link.springer.com/10.1007/BF00163878.

[S18] Lohmueller KE. The impact of population demography and selection on the genetic architecture of complex traits. PLoS genetics. 2014 may;10(5):e1004379. Available from: http://dx.plos.org/10.1371/journal.pgen.1004379.

[S19] Simons YB, Turchin MC, Pritchard JK, Sella G. The deleterious mutation load is insensitive to recent population history. Nature genetics. 2014;46(August 2013):220–4. Available from: http://www.ncbi.nlm.nih.gov/pubmed/24509481.

[S20] Lohmueller KE, Indap AR, Schmidt S, Boyko AR, Hernandez RD, Hubisz MJ, et al. Proportionally more deleterious genetic variation in European than in African populations. Nature. 2008;451(7181):994–997.

[S21] Fu W, Gittelman R, Bamshad M, Akey J. Characteristics of Neutral and Deleterious Protein-Coding Variation among Individuals and Populations. The American Journal of Human Genetics. 2014;95(4):421–436.

[S22] Do R, Balick D, Li H, Adzhubei I, Sunyaev S, Reich D. No evidence that selection has been less effective at removing deleterious mutations in Europeans than in Africans. Nature Genetics. 2015 jan;47(2):126–131. Available from: http://www.nature.com/doifinder/10.1038/ng.3186.

[S23] Peischl S, Excoffier L. Expansion load: recessive mutations and the role of standing genetic variation. Molecular Ecology. 2015 may;24(9):2084–2094. Available from: http://doi.wiley.com/10.1111/mec.13154.

[S24] Henn BM, Botigué LR, Peischl S, Dupanloup I, Lipatov M, Maples BK, et al. Distance from sub-Saharan Africa predicts mutational load in diverse human genomes. Proceedings of the National Academy of Sciences. 2016 jan;113(4):E440–E449. Available from: http://www.pnas.org/lookup/doi/10.1073/pnas.1510805112.

[S25] Kirkpatrick M, Jarne P. The Effects of a Bottleneck on Inbreeding Depression and the Genetic Load. The American Naturalist. 2000 feb;155(2):154–167. Available from: http://www.journals.uchicago.edu/doi/10.1086/303312.

[S26] Zhang XS, Hill WG. Joint Effects of Pleiotropic Selection and Stabilizing Selection on the Maintenance of Quantitative Genetic Variation at Mutation-Selection Balance. Genetics. 2002 sep;162(1):459–471. Available from: http://www.genetics.org/content/162/1/459.long.

[S27] R Core Team. R: A Language and Environment for Statistical Computing. Vienna, Austria; 2014. Available from: http://www.R-project.org/.

[S28] Fisher RA. The Genetical Theory Of Natural Selection : Fisher, R. A : Free Download & Streaming : Internet Archive. Oxford, UK; 1930. Available from: https://archive.org/details/geneticaltheoryo031631mbp.
