# Supplementary figures and images for: A Model of Compound Heterozygous, Loss-of-Function Alleles Is Broadly Consistent with Observations from Complex-Disease GWAS Datasets
Source: PLoS Genet. 2017 Jan 19;13(1):e1006573. doi: 10.1371/journal.pgen.1006573 (PMC5289629; doi:10.1371/journal.pgen.1006573)

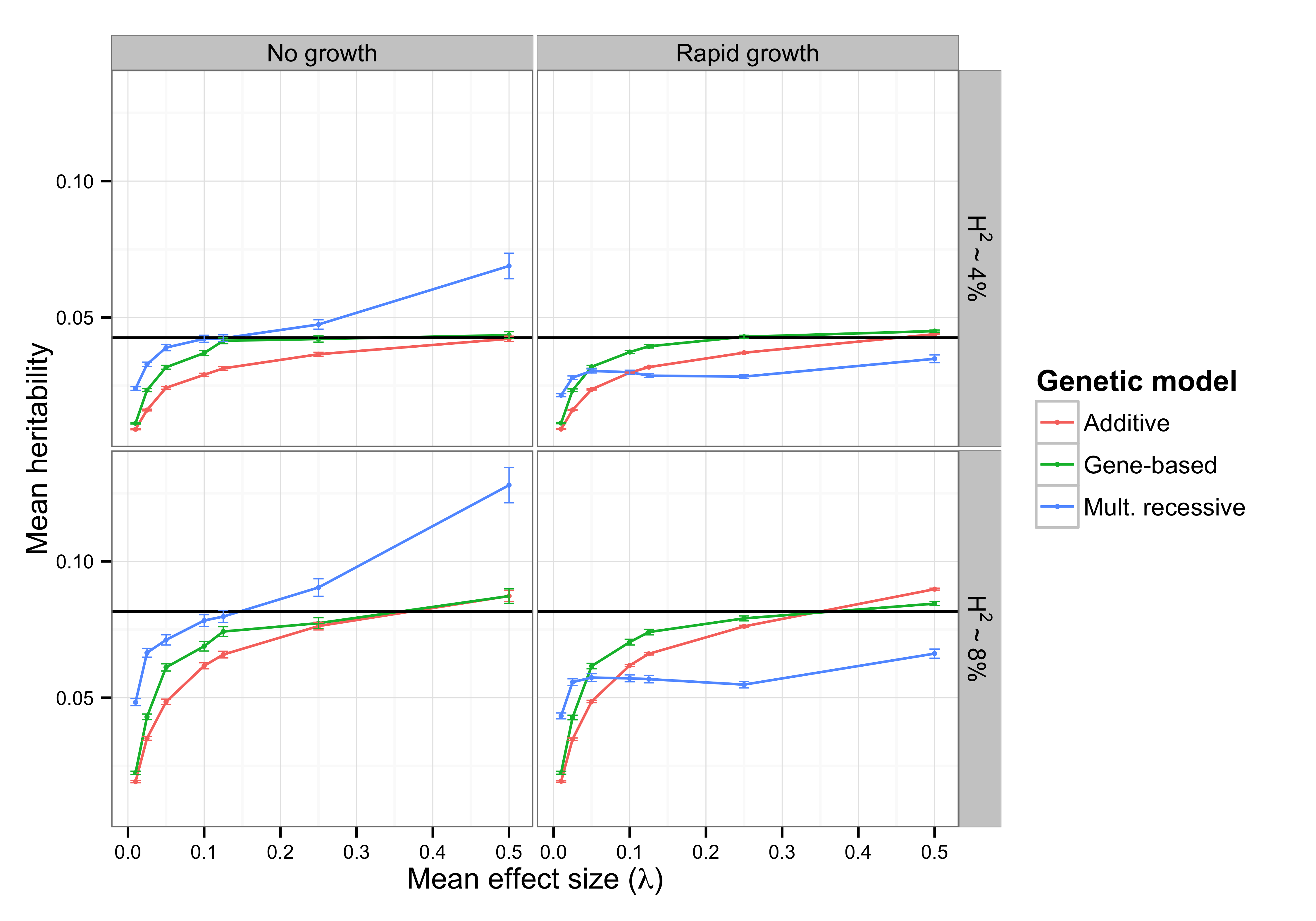

Supplement: S1 Fig — Broad-sense heritability, H2 = (VG)/(VP), as a function of λ: the mean effect size of a new deleterious mutation, as calculated explicitly from our simulated populations. Data are plotted as the mean across model replicates ± the standard error of the mean. The solid black horizontal line shows the predicted H2 under the respective house of cards approximation. The data is grouped by expected level of heritability and demographic scenario. For the additive model model, H2 ∼ 8% and H2 ∼ 4% imply environmental standard deviations of σe = 0.075 and σe = 0.011 respectively. For recessive models, H2 ∼ 8% and H2 ∼ 4% imply environmental standard deviations of σe = 0.053 and σe = 0.075 respectively. Shown are the additive co-dominant (AC), gene-based (GBR) and complete multiplicative recessive (Mult. recessive (h = 0); cMR) models. (TIFF) [file pgen.1006573.s002.tiff]

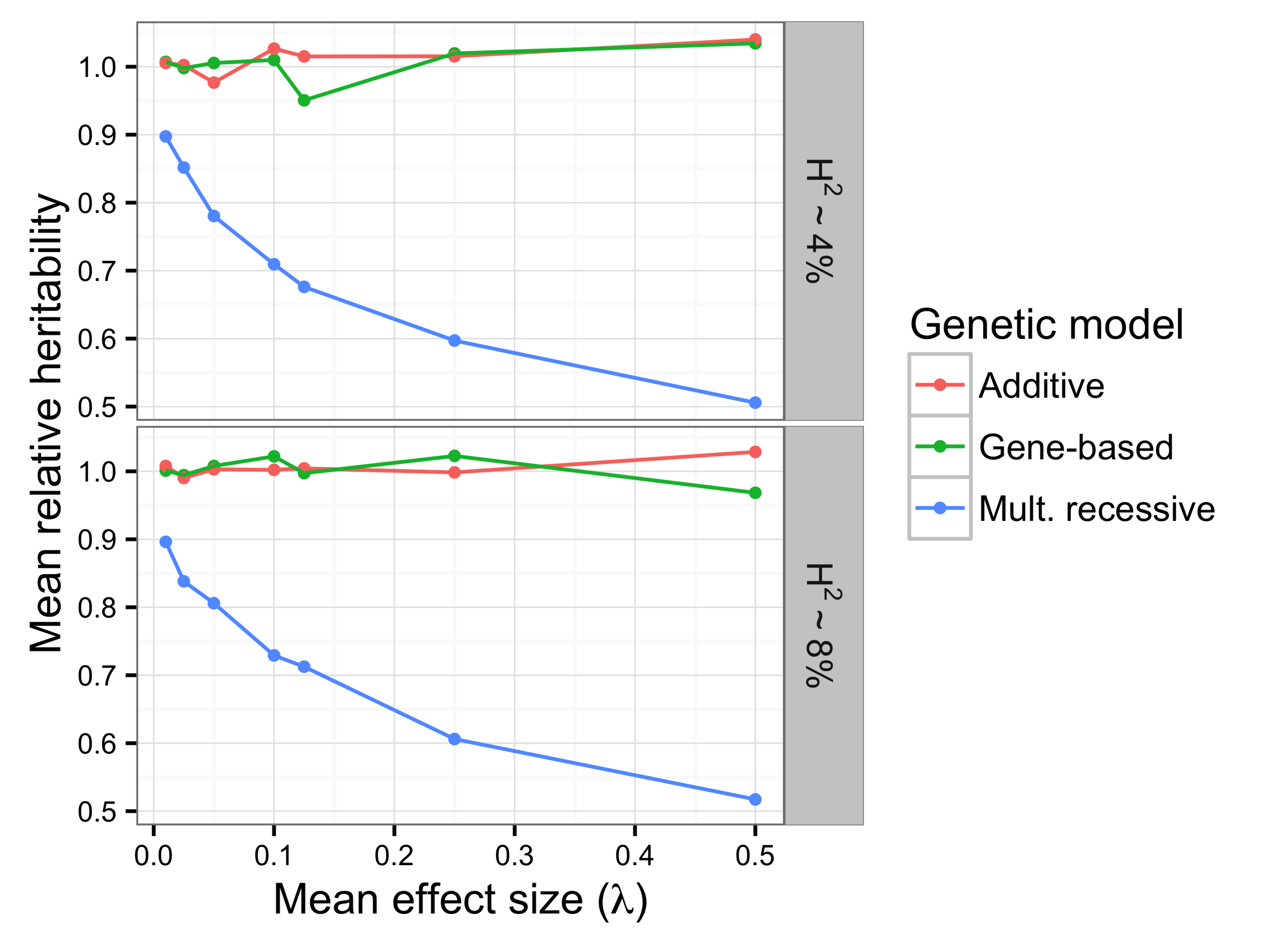

Supplement: S2 Fig — The y-axis is the ratio of mean broad-sense heritability under recent rapid growth to mean broad sense heritability for a constant-sized population, e.g. Mean[H2]growth/Mean[H2]constant. This ratio is plotted as a function of the mean effect size of causative mutations (λ). For co-dominant models, H2 ∼ 8% and H2 ∼ 4% imply environmental standard deviations of σe = 0.075 and σe = 0.011 respectively. For recessive models, H2 ∼ 8% and H2 ∼ 4% imply environmental standard deviations of σe = 0.053 and σe = 0.075 respectively. Shown are the additive co-dominant (AC), gene-based (GBR) and complete multiplicative recessive (Mult. recessive (h = 0); cMR) models. (TIFF) [file pgen.1006573.s003.tiff]

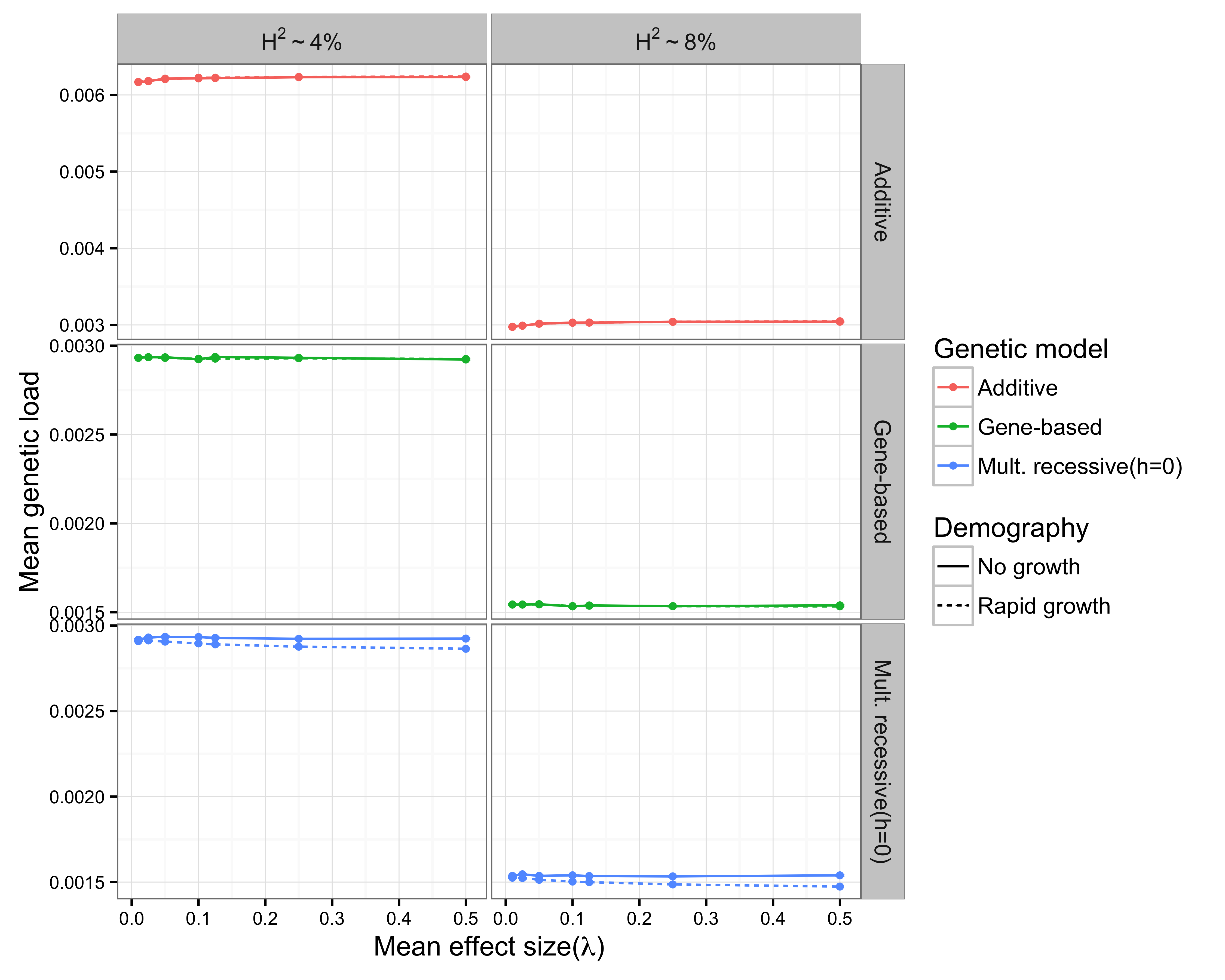

Supplement: S3 Fig — Genetic load(burden), L=wopt-w¯wopt, as a function of λ: the mean effect size of a new deleterious mutation. Data are plotted as the mean across model replicates ± the standard error of the mean. Solid curves show values for constant sized population simulations and dashed curves show values for rapid population expansion simulations. The data is grouped by expected level of heritability and genetic model. For the additive model, H2 ∼ 8% and H2 ∼ 4% imply environmental standard deviations of σe = 0.075 and σe = 0.011 respectively. For recessive models, H2 ∼ 8% and H2 ∼ 4% imply environmental standard deviations of σe = 0.053 and σe = 0.075 respectively. Note the scales of y-axis for each plot. Shown are the additive co-dominant (AC), gene-based (GBR) and complete multiplicative recessive (Mult. recessive (h = 0); cMR) models. (TIFF) [file pgen.1006573.s004.tiff]

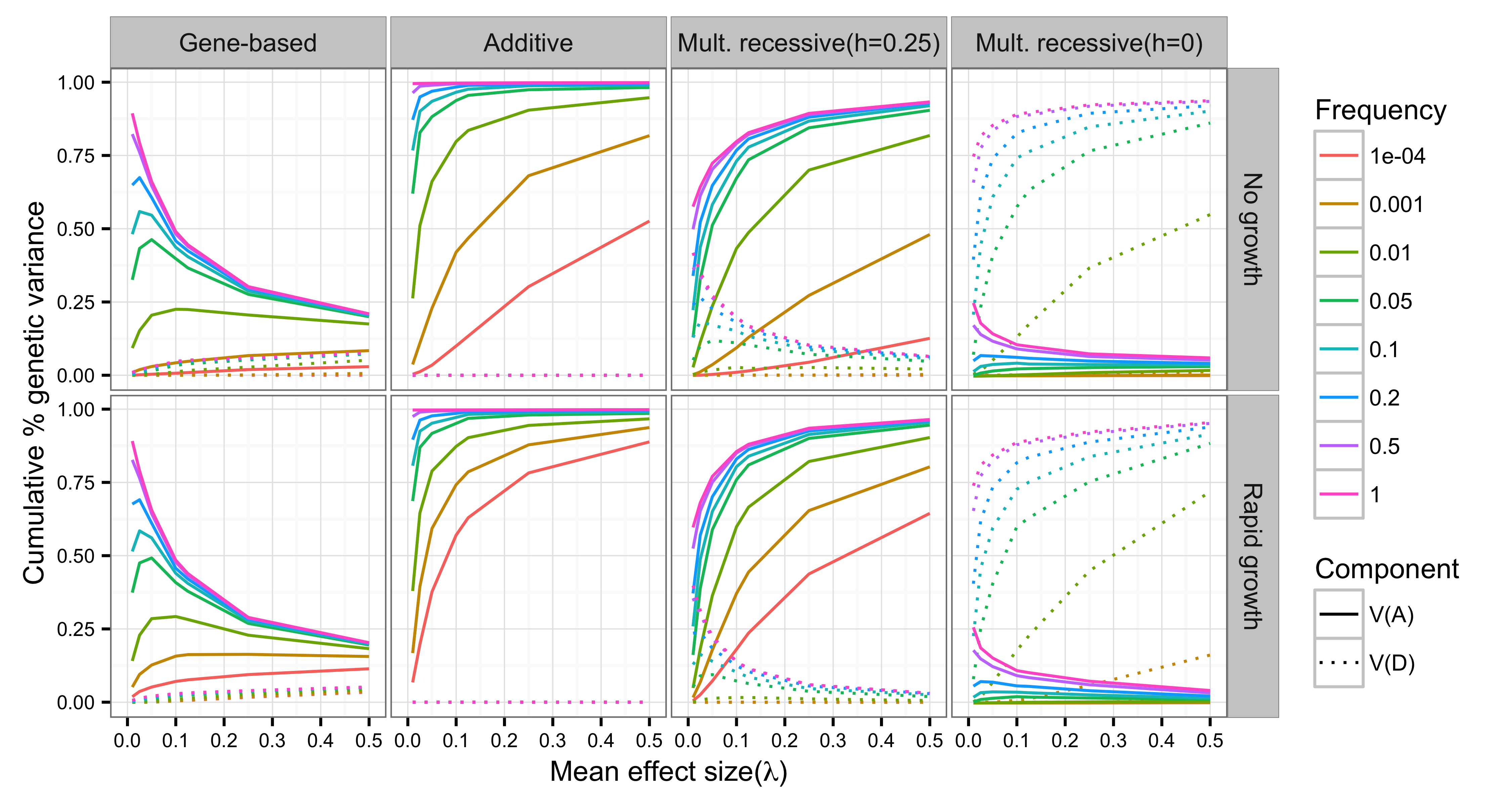

Supplement: S4 Fig — The percent of cumulative genetic variance explained by additive and dominance effects of variants with frequency less than or equal to a series of frequency values over λ. Shown here are the gene-based (GBR), additive co-dominant (AC), incomplete multiplicative recessive (Mult. recessive (h = 0.25); iMR) and complete multiplicative recessive (Mult. recessive (h = 0);cMR) models. Solid lines show the additive variance alone and dotted lines show the combined additive and dominance variance. All data shown are for models where H2 ∼ 0.08. These particular results are robust to changes H2 when VG is not changed, as is the case here. The additive and dominance genetic variance is estimated by the adjusted r2 of the regression of all markers (and their corresponding dominance encoding) with MAF ≤ x onto total genotypic value (see methods for details); data are displayed as the mean of 250 simulation replicates. For each frequency level we calculated the r2 of a linear regression of genotypes of markers with frequency below that level on to total genetic value and plot it against λ: the mean effects size of a new deleterious mutation. The data are displayed as a mean across model replicates. (TIFF) [file pgen.1006573.s005.tiff]

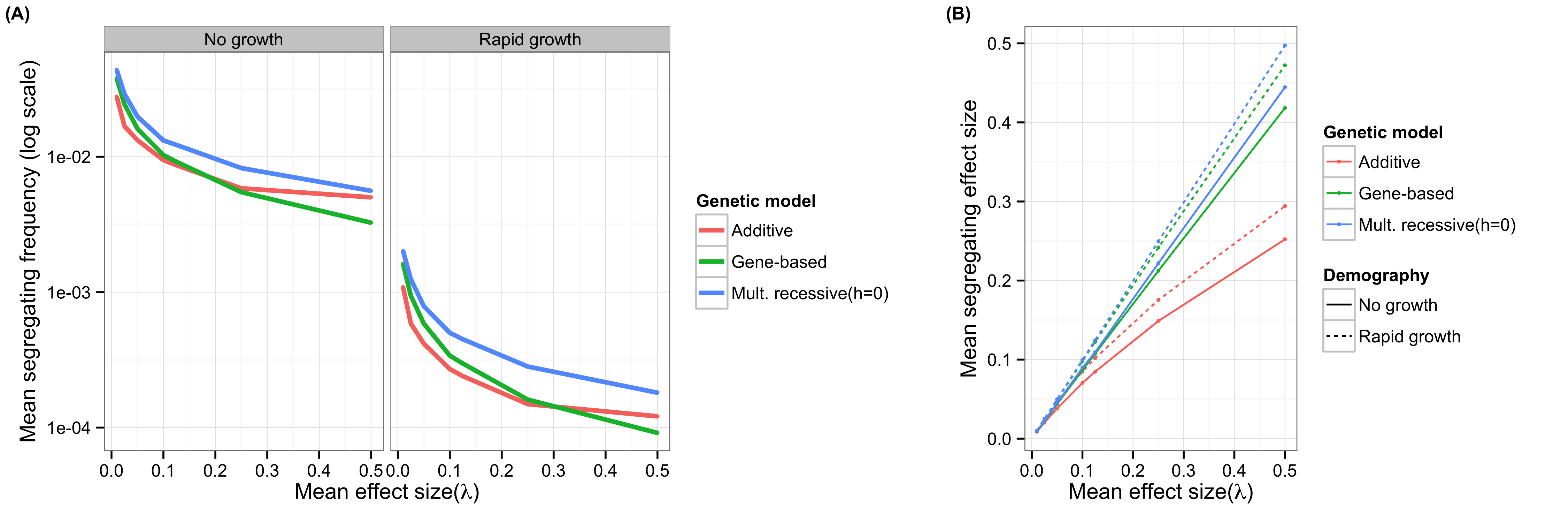

Supplement: S5 Fig — A) The mean frequency and B) mean effect size of a segregating risk variant over λ. Note they log10 y-axis scale in A. The mean effect size is the value pulled from the exponential distribution with mean λ, not the fitness effect or the quantitative genetic effect size. The data are calculated for all risk mutations segregating in the simulated populations. Data are plotted as the mean across model replicates. For visual clarity, standard errors are not shown. In panel A, the standard error bars overlap zero under rapid population growth. The data for mean frequency are grouped by demographic scenario; the left panel shows values for constant sized population, the right panel shows values for the rapidly expanded populations. For mean effect size plots the solid curves show the constant sized population data and the dashed curves show the data for the rapidly expanded populations. Shown are the additive co-dominant (AC), gene-based (GBR) and complete multiplicative recessive (Mult. recessive (h = 0); cMR) models. (TIFF) [file pgen.1006573.s006.tiff]

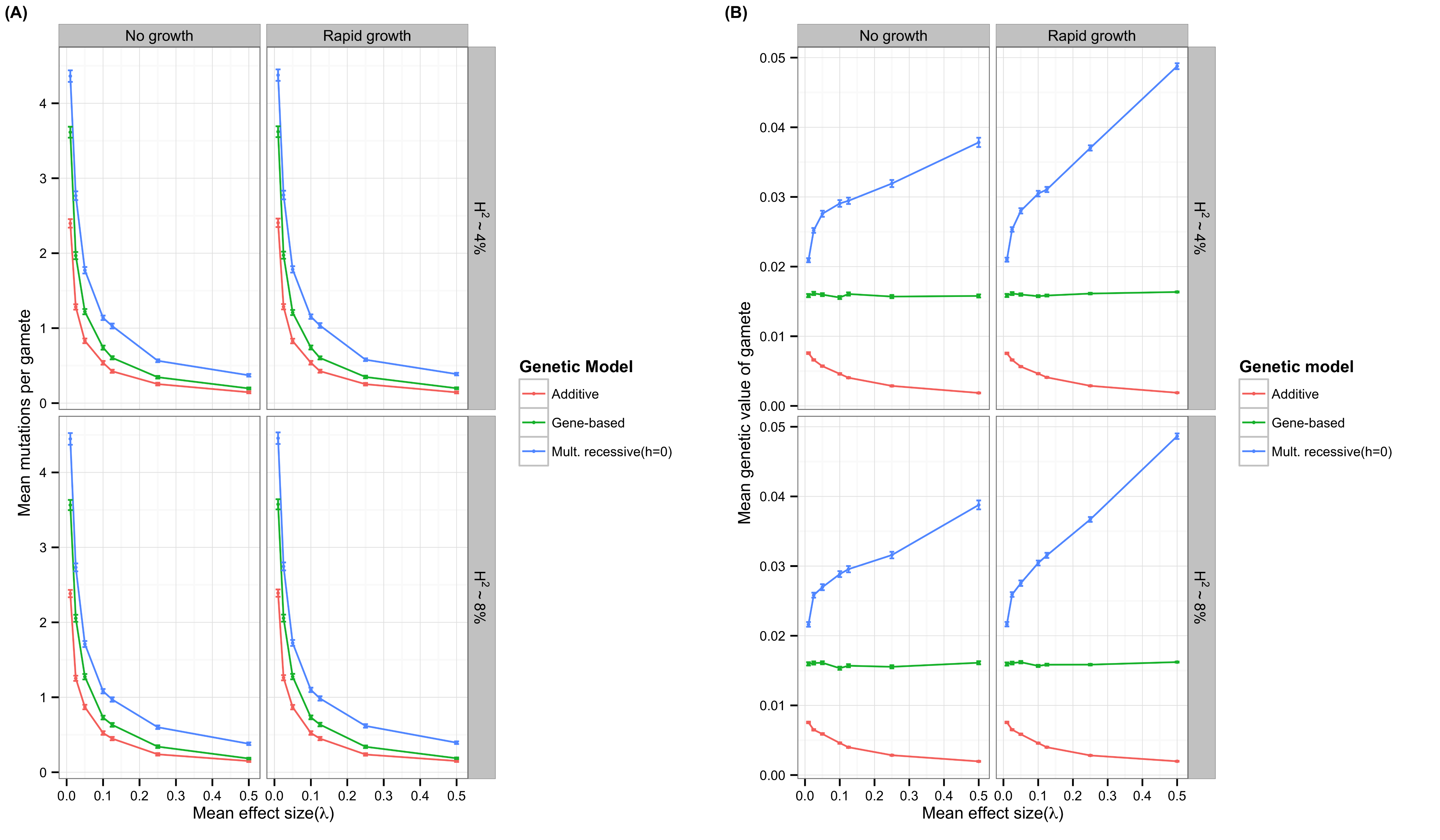

Supplement: S6 Fig — A) The mean number of deleterious mutations per gamete in the population as a function of λ: the mean effect size of new causative mutation. The data plotted as mean over simulation replicates ±se. The data are calculated for the entire simulated population. B) The mean genetic value of a gamete, i.e. the average sum of mutational effect sizes on a gamete as a function of λ. Data are plotted as the mean across model replicates ± the standard error of the mean. In the case the gene-based recessive model, this value is also the expected value of the mean phenotype and is accurate within the sampling variance of the mean environmental variate and random pairing of gametes in diploid. Shown are the additive co-dominant (AC), gene-based (GBR) and complete multiplicative recessive (Mult. recessive (h = 0); cMR) models. (TIFF) [file pgen.1006573.s007.tiff]

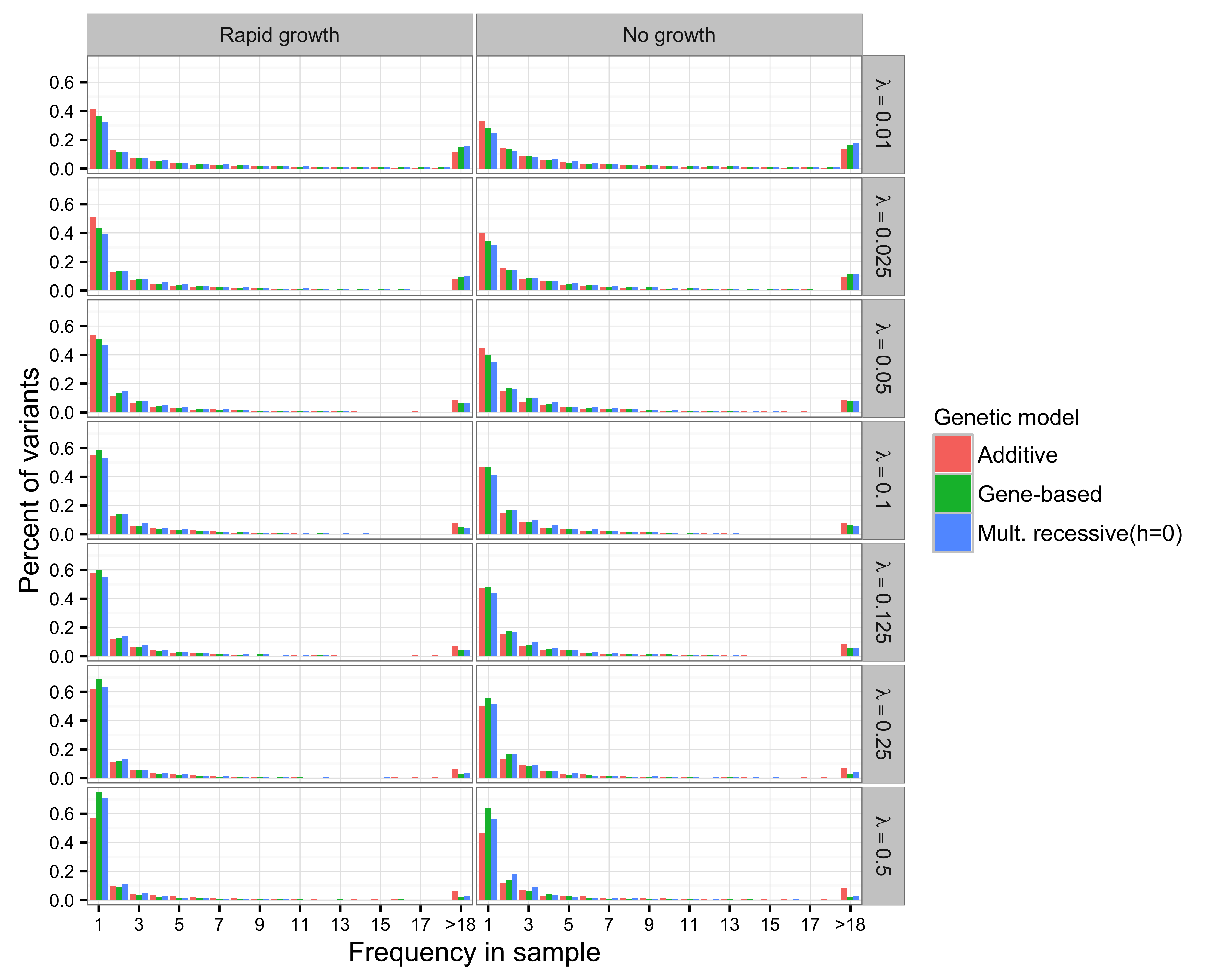

Supplement: S7 Fig — For a sample n = 100 individuals, the relative site frequency spectrum is calculated as the proportion (y-axis) of all polymorphic sites which belong to each frequency class (x-axis). Sites with frequency was above 18 were grouped, into one category to improve visualization. The data are grouped by λ, the mean effect size of a new risk mutation, and the demographic scenario. Data shown are for simulations in which the predicted broad sense heritability is H2 ∼ 8%. Plotted values are the mean proportion across simulation replicates. Shown are the additive co-dominant (AC), gene-based (GBR) and complete multiplicative recessive (Mult. recessive (h = 0); cMR) models. (TIFF) [file pgen.1006573.s008.tiff]

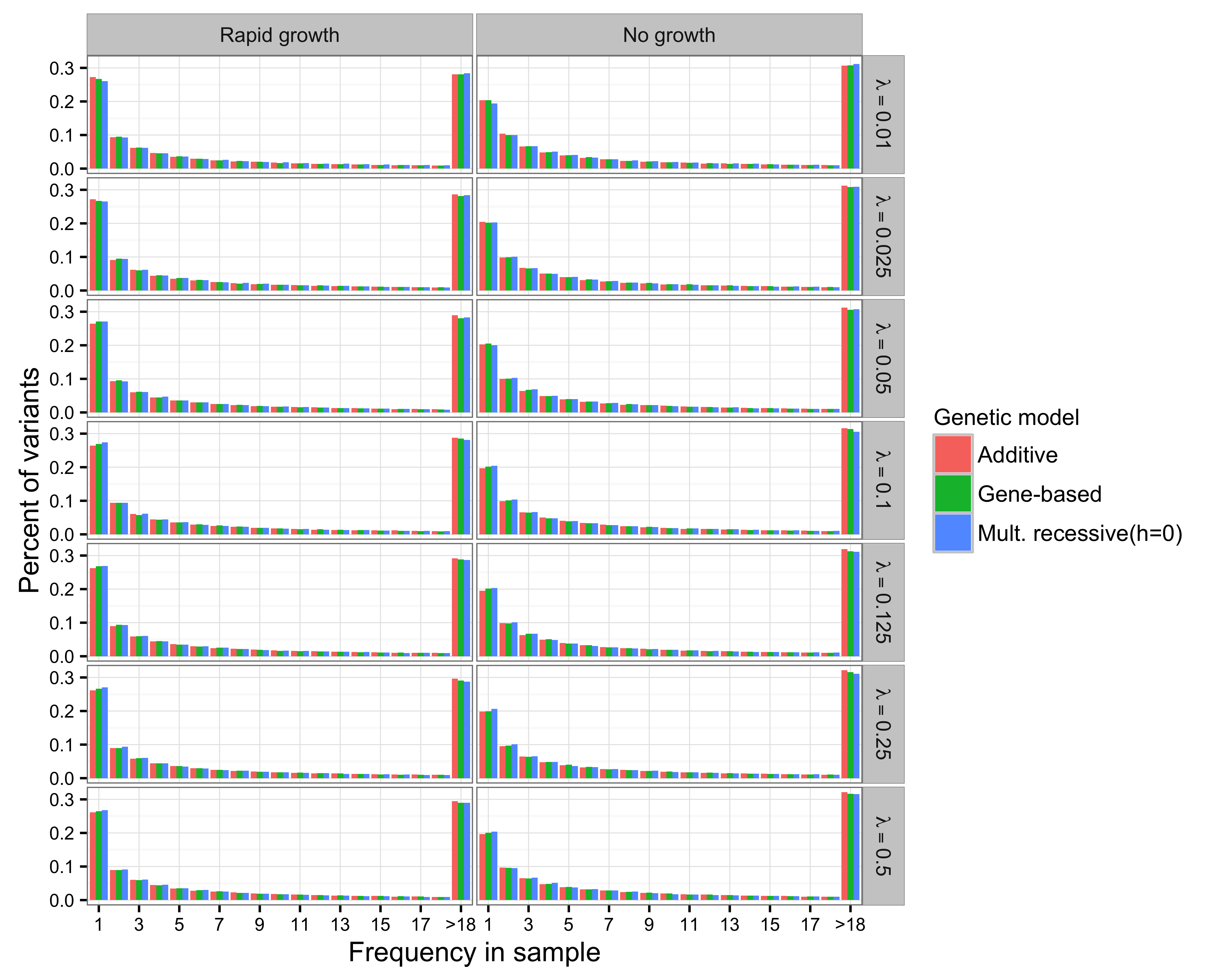

Supplement: S8 Fig — For a sample n = 100 individuals, the relative site frequency spectrum is calculated as the proportion (y-axis) of all polymorphic sites which belong to each frequency class (x-axis). Sites with frequency was above 18 were grouped into one category to improve visualization. The data are grouped by λ, the mean effect size of a new risk mutation, and the demographic scenario. Data shown are for simulations in which the predicted broad sense heritability is H2 ∼ 8%. Plotted values are the mean proportion across simulation replicates. Shown are the additive co-dominant (AC), gene-based (GBR) and complete multiplicative recessive (Mult. recessive (h = 0); cMR) models. (TIFF) [file pgen.1006573.s009.tiff]

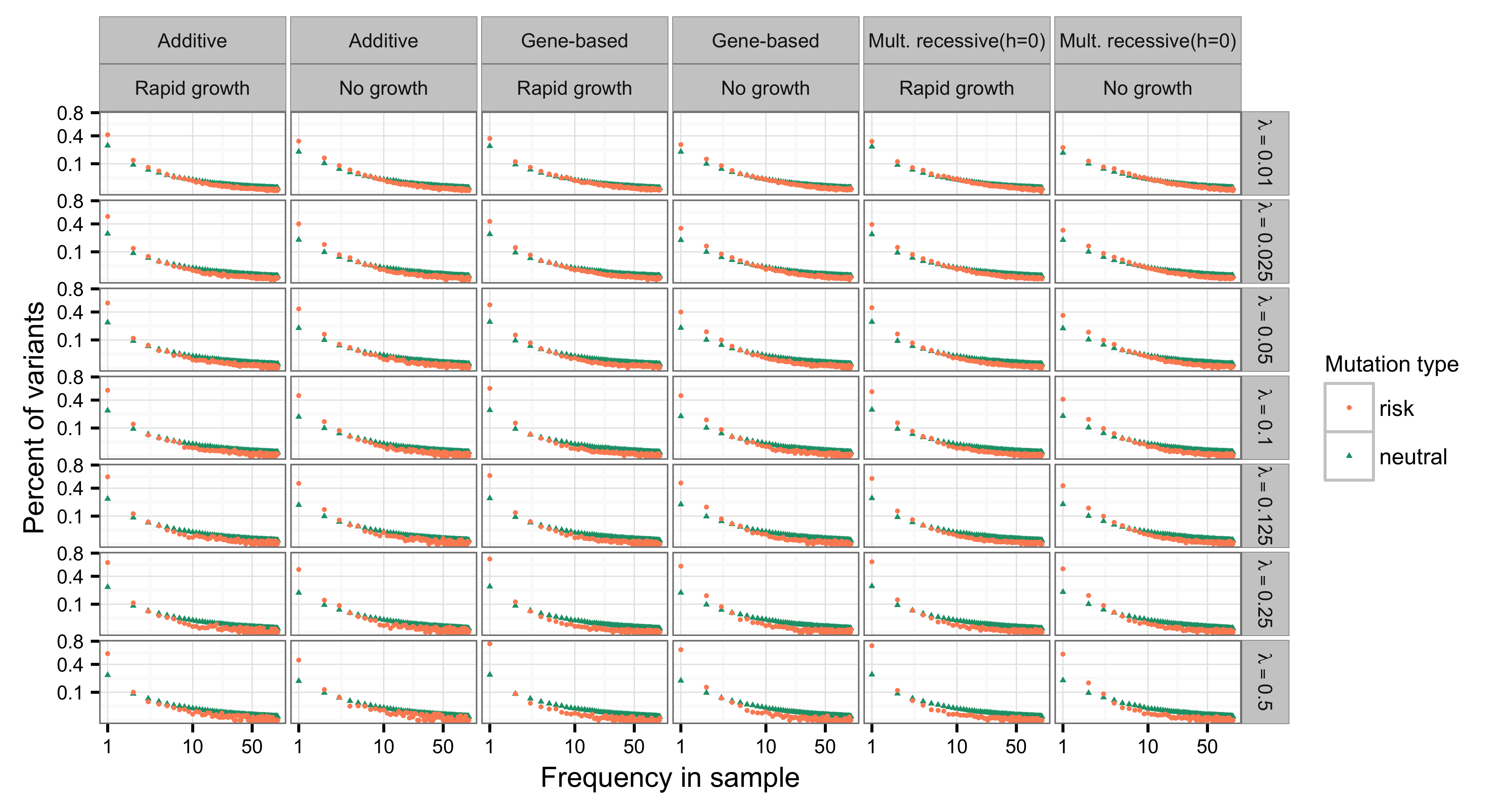

Supplement: S9 Fig — For a sample n = 100 individuals, the relative site frequency spectrum is calculated as the proportion (y-axis) of all polymorphic sites which belong to each frequency class (x-axis). Neutral variants are in orange and risk variants are shown in green. Y-axis is on a square-root scale and X-axis is on a log10 scale to improve visualization. The data are grouped by λ, the mean effect size of a new risk mutation, the demographic scenario and genetic model. Data shown are for simulations in which the predicted broad sense heritability is H2 ∼ 8%. Plotted values are the mean proportion across simulation replicates. Shown are the additive co-dominant (AC), gene-based (GBR) and complete multiplicative recessive (Mult. recessive (h = 0); cMR) models. (TIFF) [file pgen.1006573.s010.tiff]

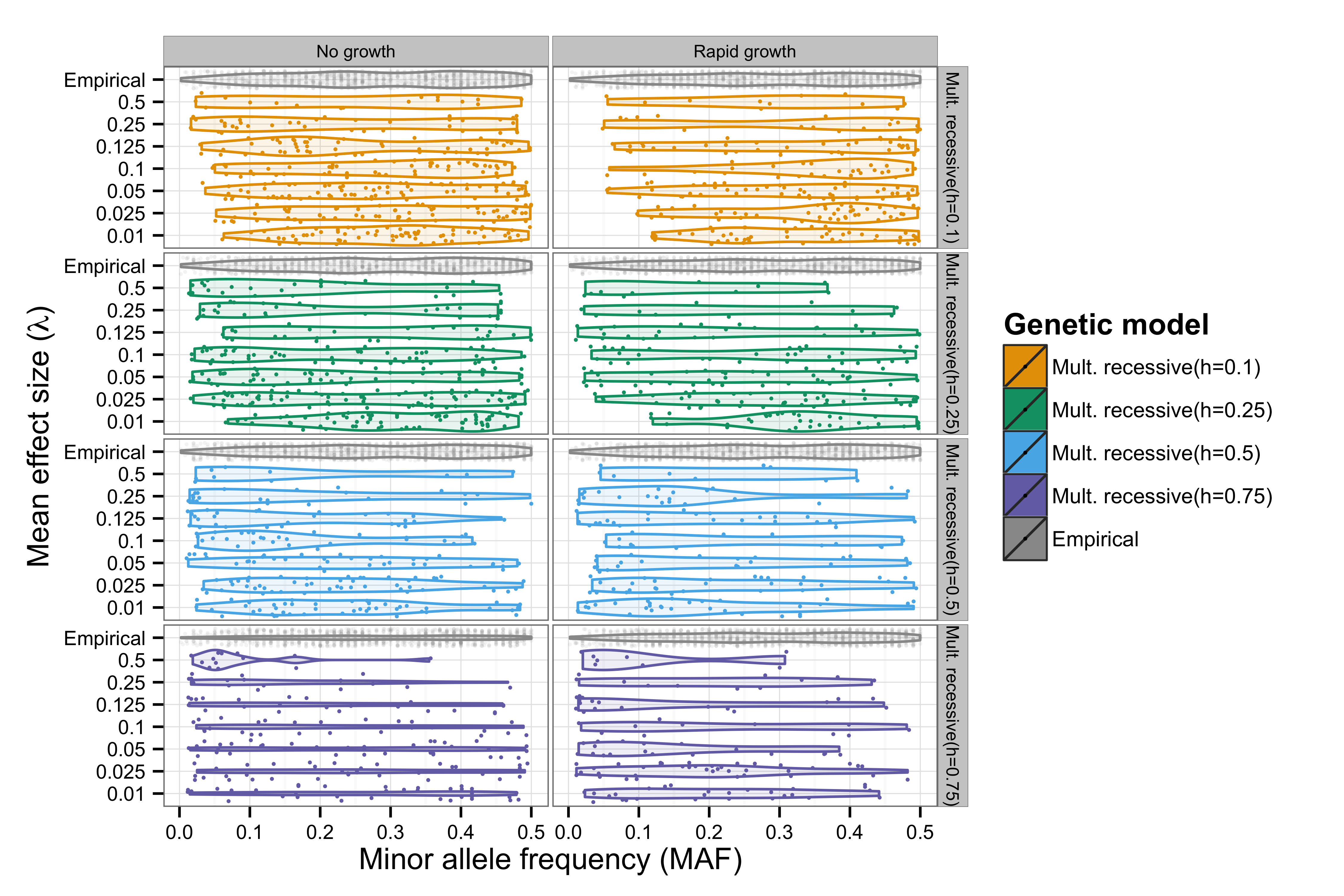

Supplement: S10 Fig — Horizontal violin plots depict the distribution of minor allele frequencies (MAF) of the most strongly associated single marker in a GWAS. Individual hits are plotted as translucent points and jittered to provide a sense of the total number and density of hits. Each panel contains simulated data pooled across model replicates for each value of λ, with empirical data for comparison. The degree of dominance h was varied from 0.1 to 0.75; perfect co-dominance here is h = 1. Empirical data were downloaded from the NHGRI-EBI GWAS database (http://www.ebi.ac.uk/gwas/) on 02/03/2015, diseases and inclusion criteria are as in [26]. In cases where more than one marker was tied for the lowest p-value, one was chosen at random. Simulated data were subjected to ascertainment sampling such that the MAF distribution of all markers on the simulated genotyping chip was uniform. Specific information regarding the empirical data can be obtained in S1 Table. (TIFF) [file pgen.1006573.s011.tiff]

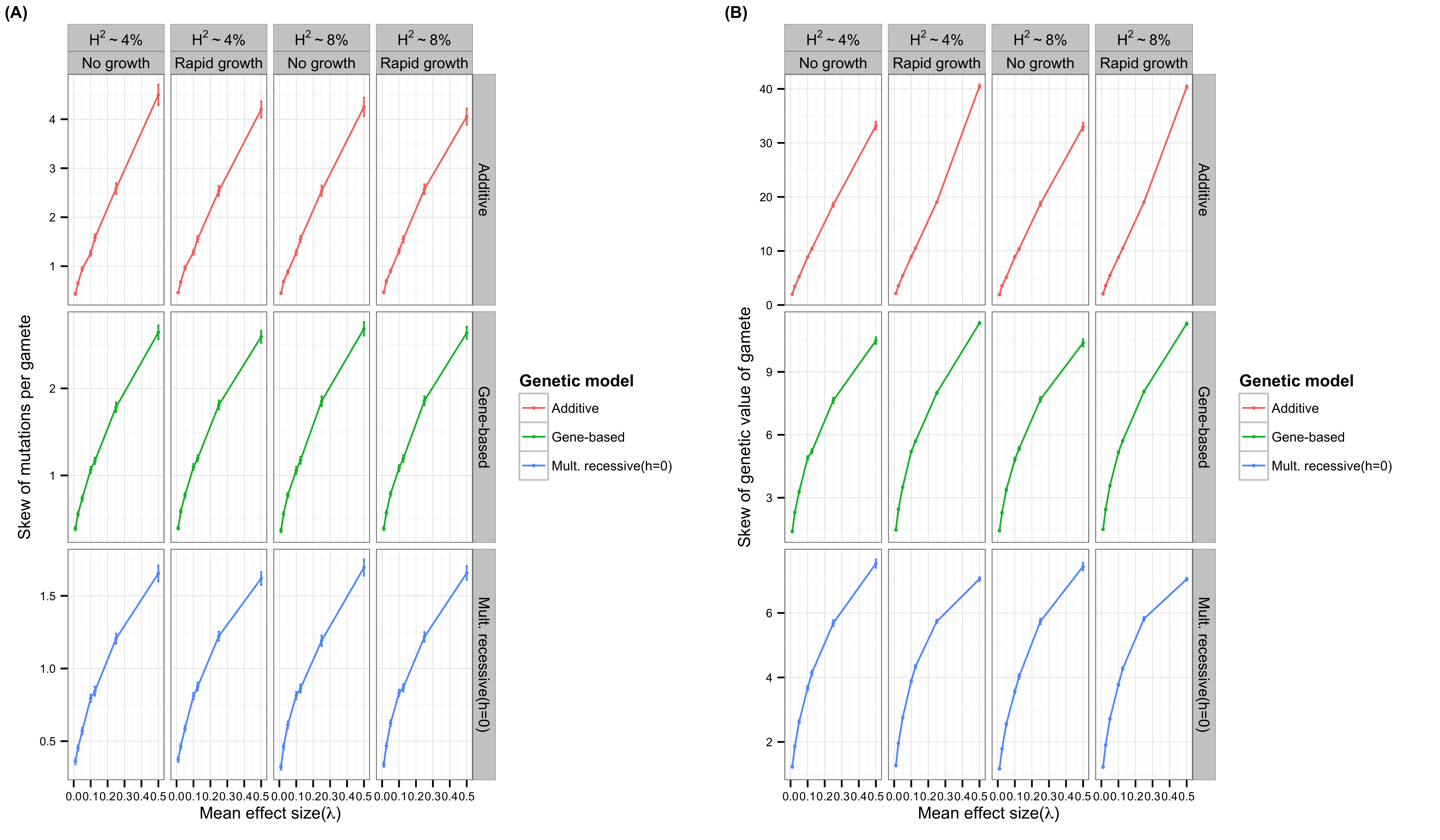

Supplement: S11 Fig — The skewness A) the number of mutations per gamete and B) the genetic value (sum of mutational effects) of a gamete over λ. The data are calculated for all risk mutations segregating in the simulated populations. Moments were calculated using the boost C++ statistical accumulators library. Data are plotted as the mean across model replicates ± the standard error of the mean. Shown are the additive co-dominant (AC), gene-based (GBR) and complete multiplicative recessive (Mult. recessive (h = 0); cMR) models. (TIFF) [file pgen.1006573.s012.tiff]

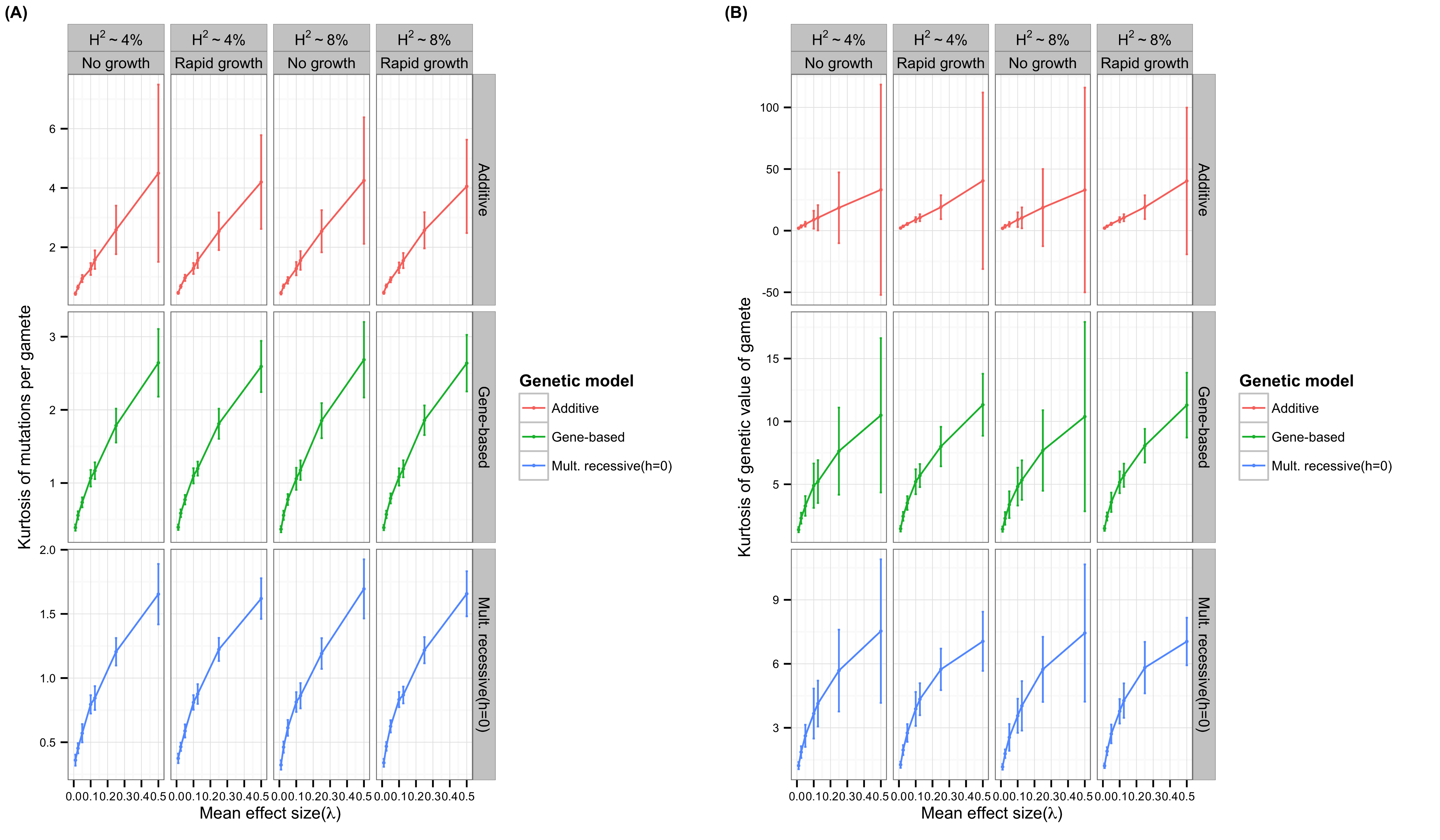

Supplement: S12 Fig — The kurtosis A) the number of mutations per gamete and B) the genetic value (sum of mutational effects) of a gamete over λ. The data are calculated for all risk mutations segregating in the simulated populations. Moments were calculated using the boost C++ statistical accumulators library. Data are plotted as the mean across model replicates ± the standard error of the mean. Shown are the additive co-dominant (AC), gene-based (GBR) and complete multiplicative recessive (Mult. recessive (h = 0); cMR) models. (TIFF) [file pgen.1006573.s013.tiff]

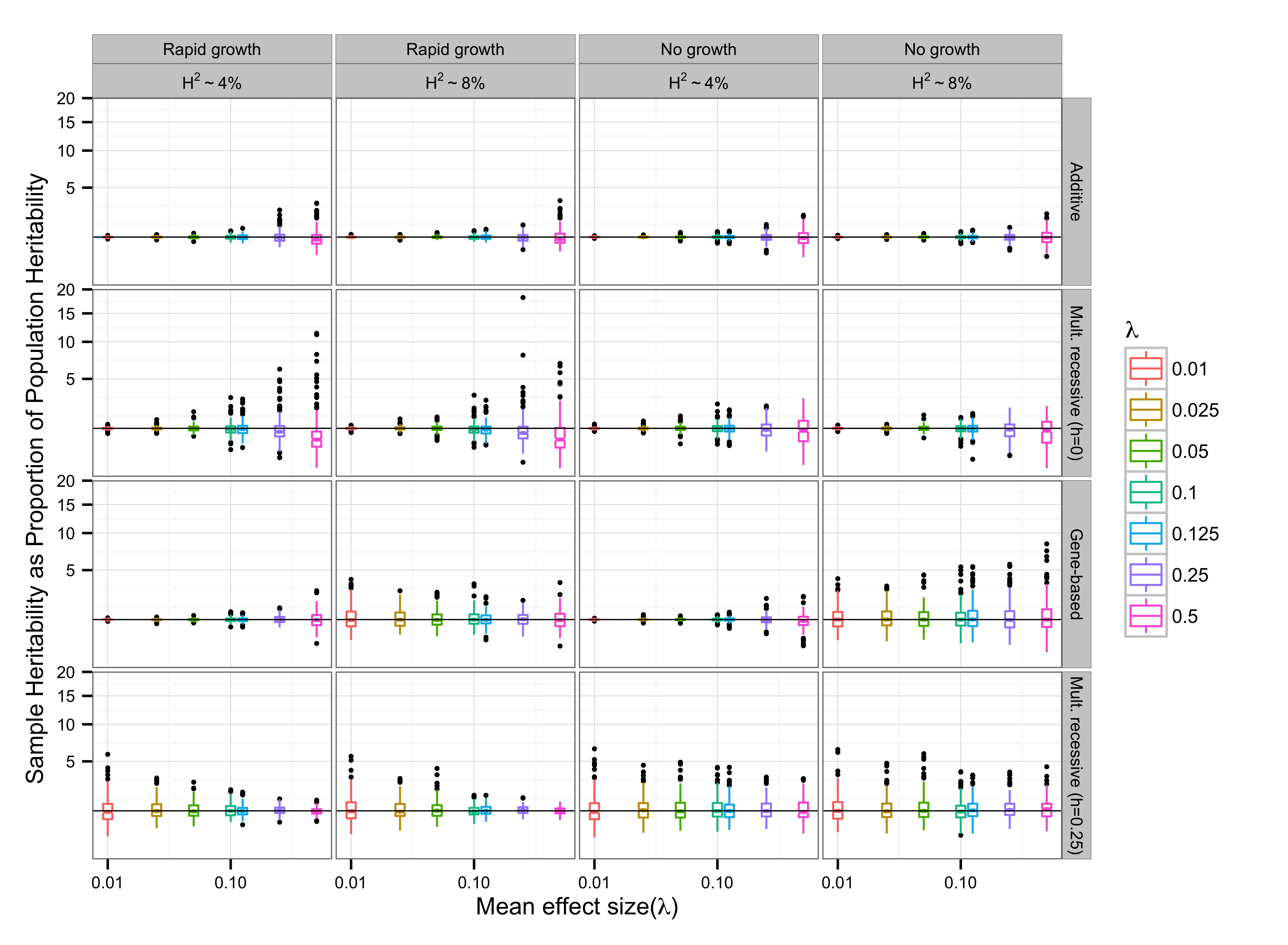

Supplement: S13 Fig — Broad-sense heritability in a population sample of 6000 as a proportion of population wide broad-sense heritability. Data are grouped by demographic scenario, model and λ. The arbitrary dominance coefficient is parameterized such that h = 0 is complete recessivity, h = 1 would be exact co-dominance and h = 2 would be complete dominance. Multiplicative recessive (MR) models shown are only for h = 0.25. (TIFF) [file pgen.1006573.s014.tiff]

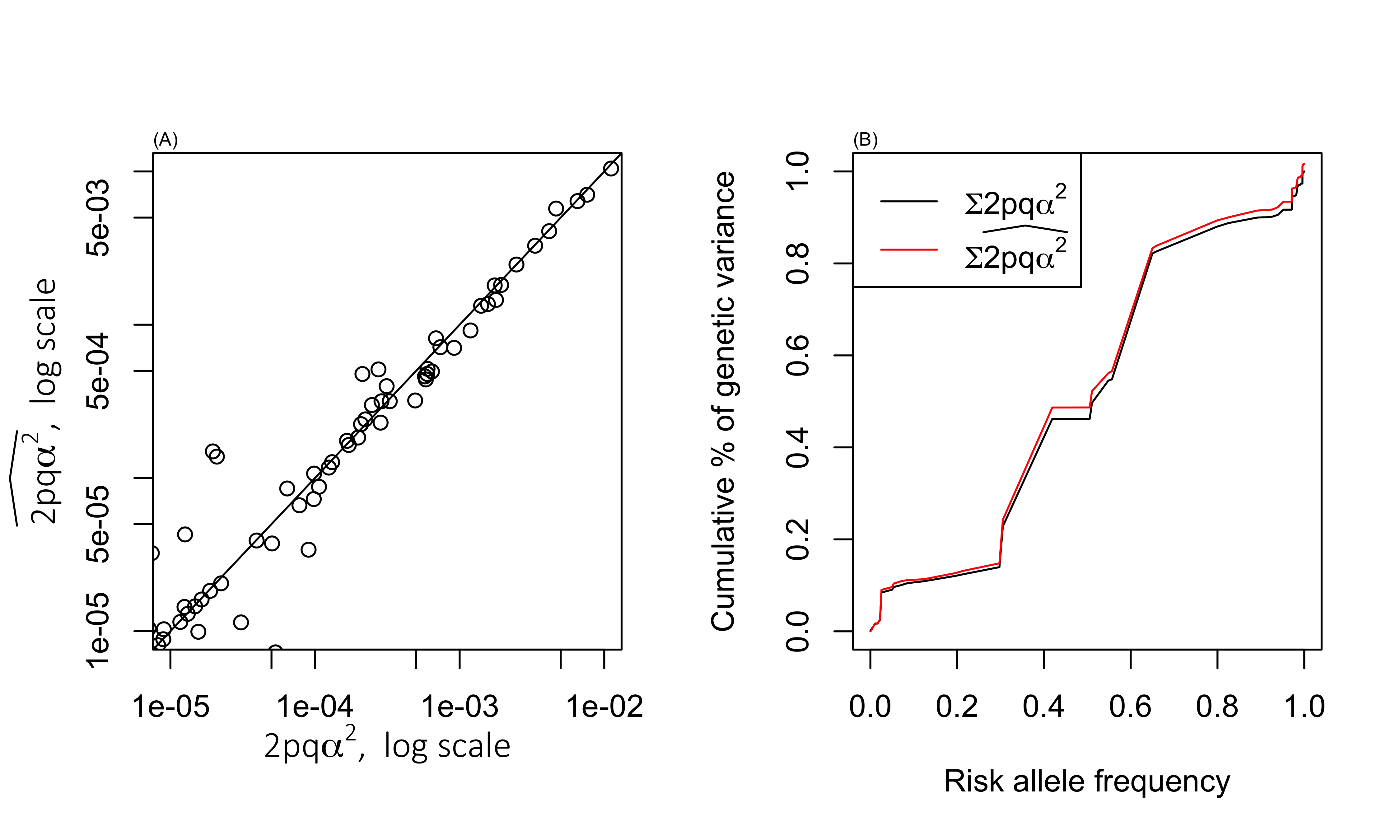

Supplement: S14 Fig — (A) Regression estimates of variance explained by markers versus the classical formula 2pqα2. (B) Cumulative percent of variance explained across the risk allele frequency, based on regression estimates and classical formula. 1000 unlinked markers were simulated with effects drawn from an exponential distribution with mean 0.1 and population frequencies drawn from the neutral Wright-Fisher allele frequency distribution. Sample data for 5000 individuals were then generated by sampling genotypes at each marker based on its allele frequency. We plot the regression estimate of variance explained by each marker against Fisher’s classic result [9]: VG = 2pqα2. (TIFF) [file pgen.1006573.s015.tiff]

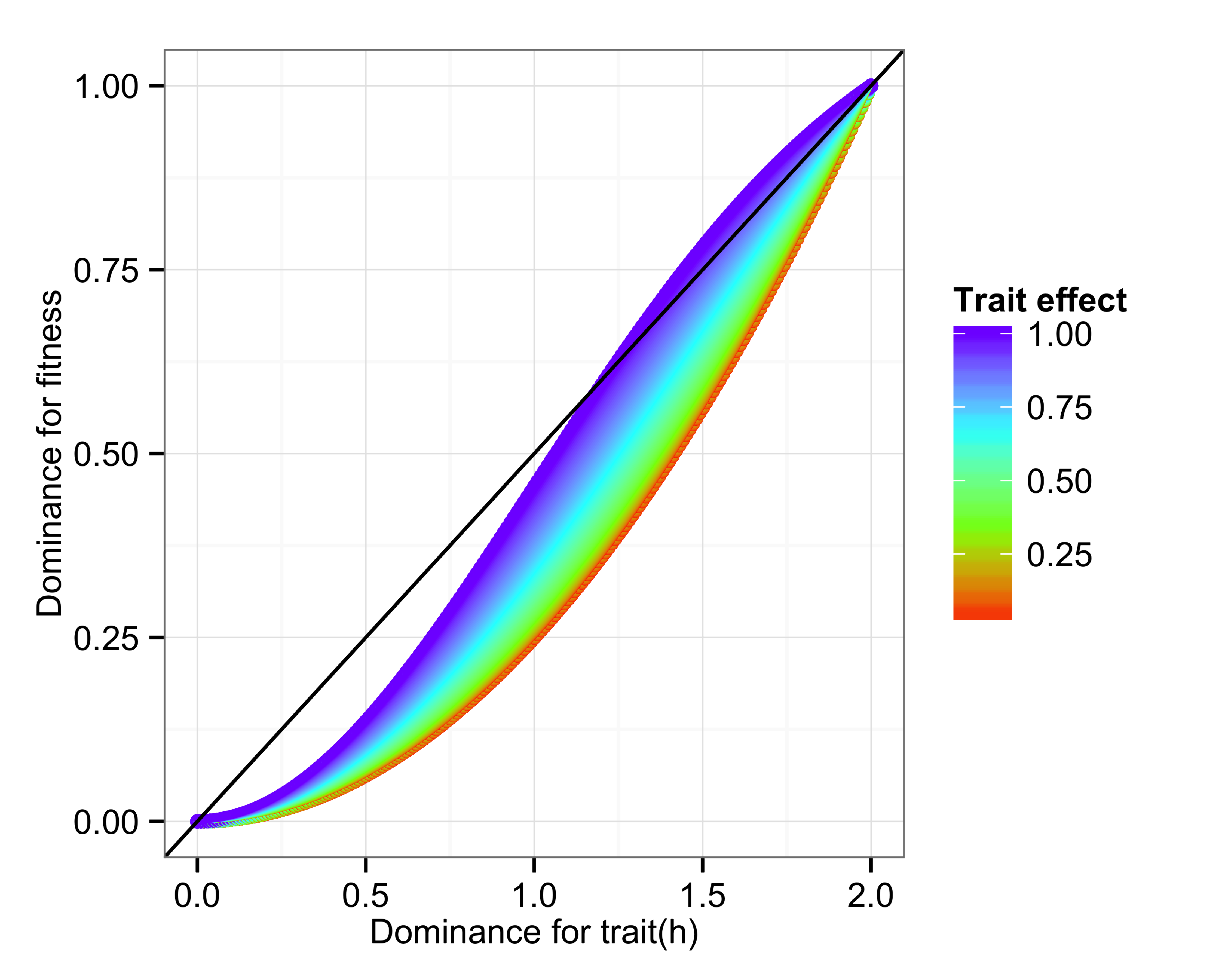

Supplement: S15 Fig — The dominance of fitness effects, shetshom, as a function of the dominance for trait effects, h. Values are based on idealized fitness effects of a mutation on a previously unaffected genetic background. The the relationship between fitness and trait dominance is influenced by the trait effect size. We varied trait effect sizes from 0.01 to 1, and values are colored based on the trait effect. (TIFF) [file pgen.1006573.s016.tiff]

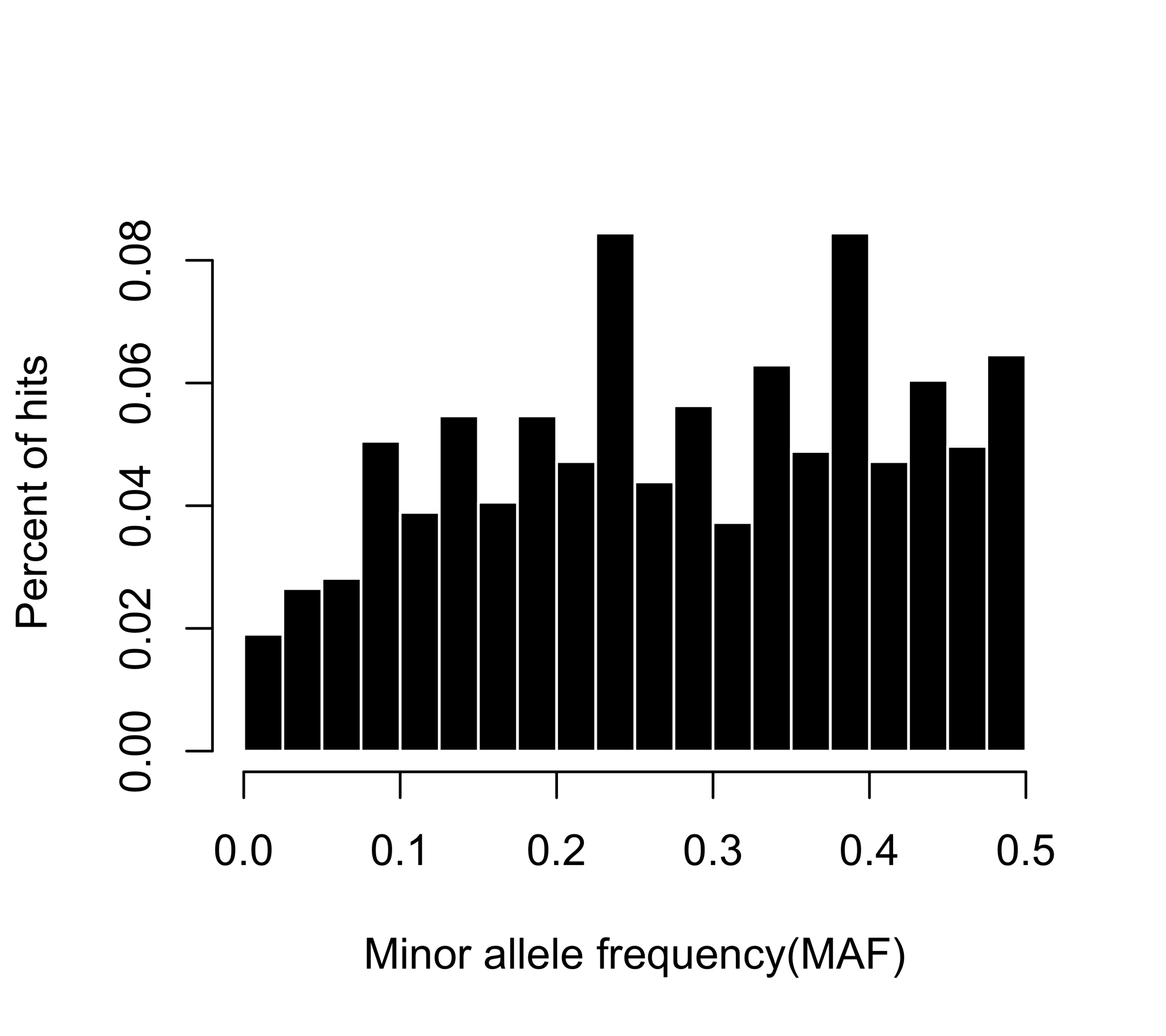

Supplement: S16 Fig — Histogram GWAS hits (n = 1208) obtained from the NHGRI-EBI GWAS database for disease discussed in [26]. Data are described in S1 Table. (TIFF) [file pgen.1006573.s017.tiff]

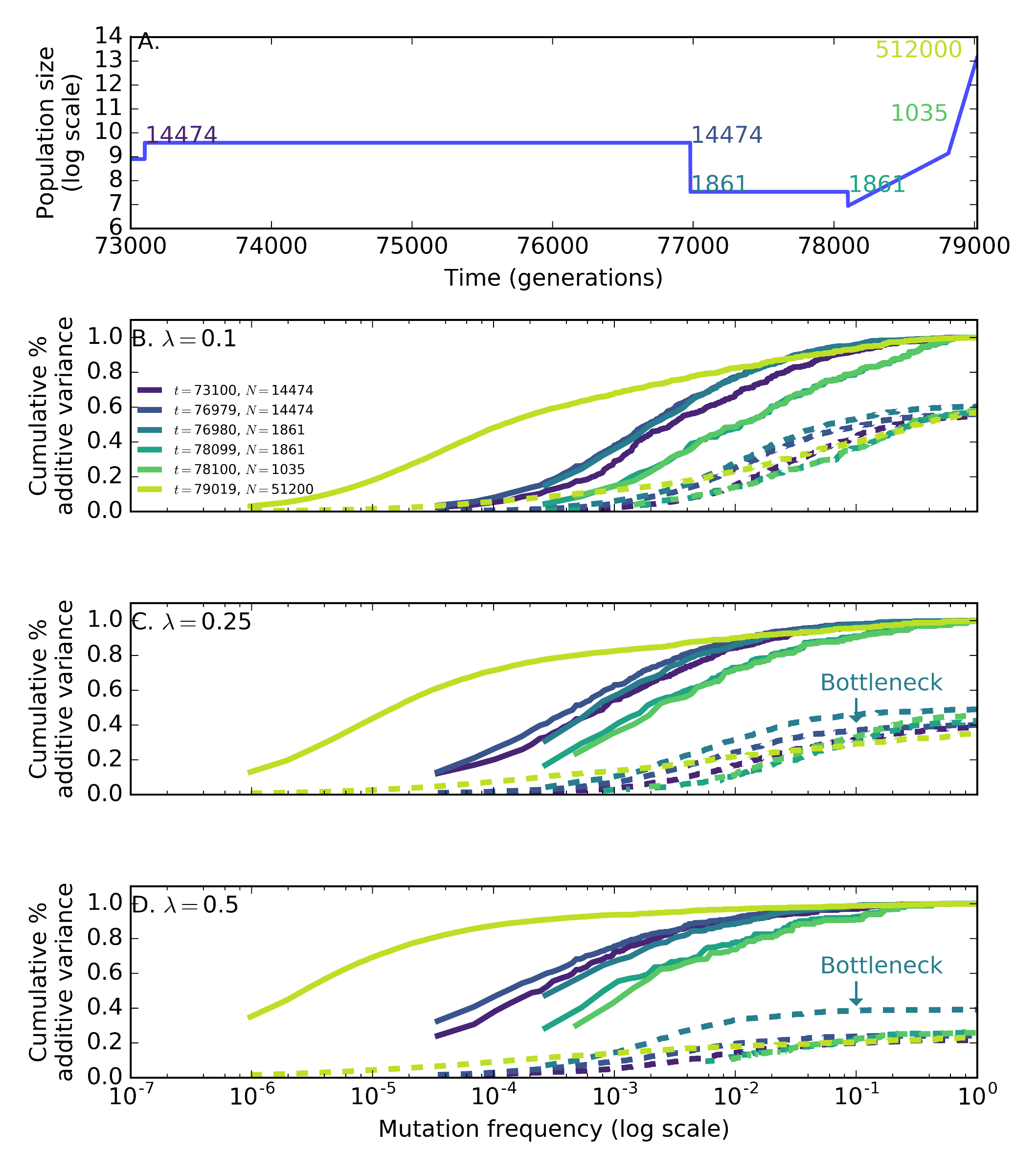

Supplement: S17 Fig — (A) Population size change over time. Colored numbers represent population sizes at different times where we estimated the cumulative additive genetic variance (VA) as a function of allele frequency using regression (see Materials and Methods). These time points represent key changes in population size in this model. (B-D) Estimated cumulative VA as a function of frequency for three different mean effect sizes (λ ∈ 0.1,0.25,0.5). Solid lines are the standard additive model. Dashed lines are the GBR model of [36]. For all time points, the same total percent of variance is explained, with the exception of the line labelled “bottleneck”. For larger effect sizes under the GBR model, the bottleneck increases the total VA explained by all mutations. This effect is, however, short lived, and disappears by the end of the epoch defined by N = 1,861. This result is consistent with transient increases in variation under recessive models reported by [21]. (TIFF) [file pgen.1006573.s018.tiff]

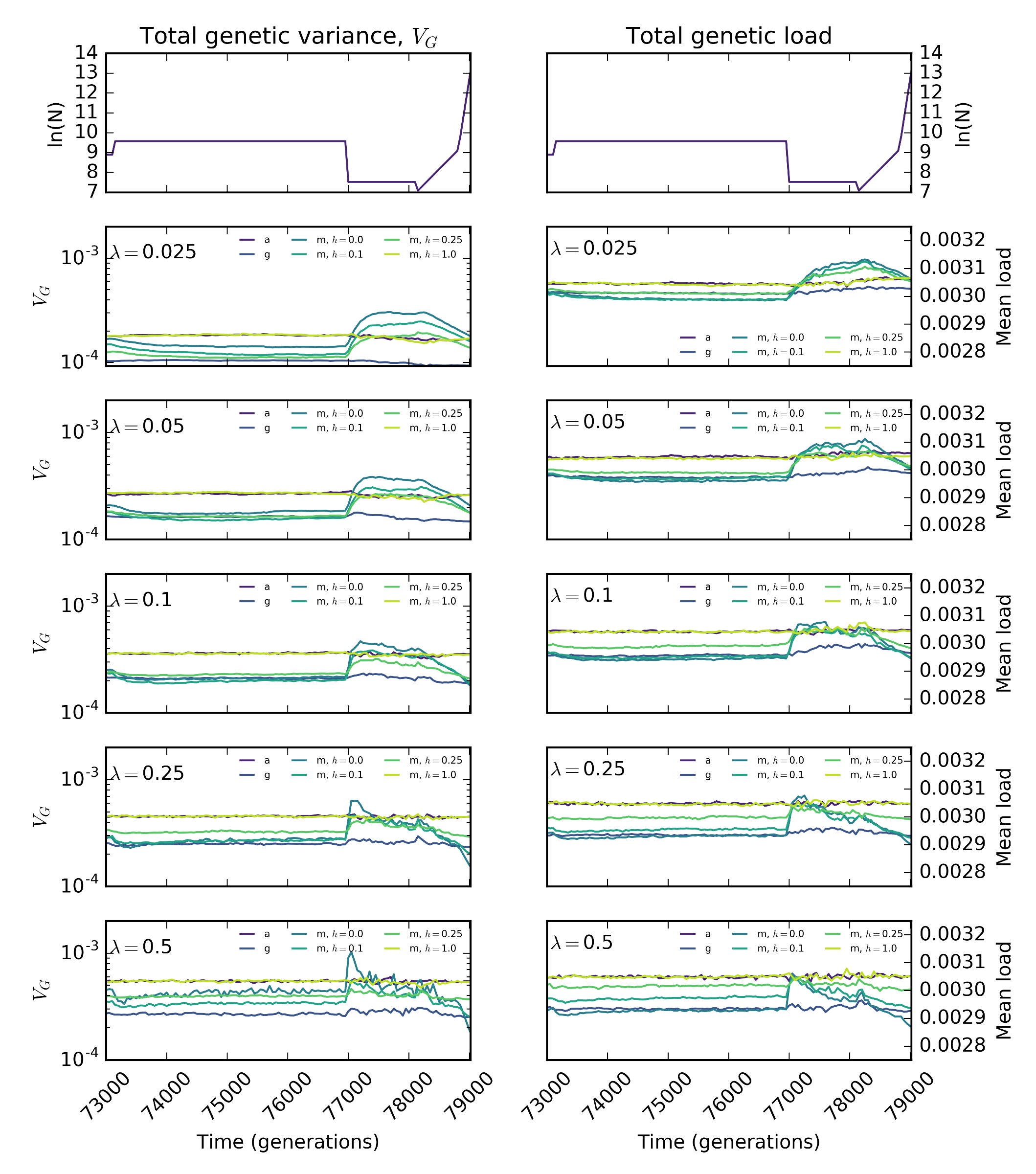

Supplement: S18 Fig — The left column of panels shows how VG changes over time under this model. The right column shows how the mean number of deleterious mutations per individual changes. The models shown are: a = additive, g = GBR, and m = multiplicative with varying degrees of dominance (h). The main difference is between additive models (a or m with h = 1.0) and recessive models (g or m with small h). The former models are largely insensitive to changes in N, while the recessive models show transient increases in VG and “load” immediately following a bottleneck (consistent with [21]). However, at the final time point representing the “modern European population”, all mean VG is ≈ 4μ for additive models and ≈ 2μ for recessive models [71, 89], and recessive models show larger loads as expected [21]. (TIFF) [file pgen.1006573.s019.tiff]

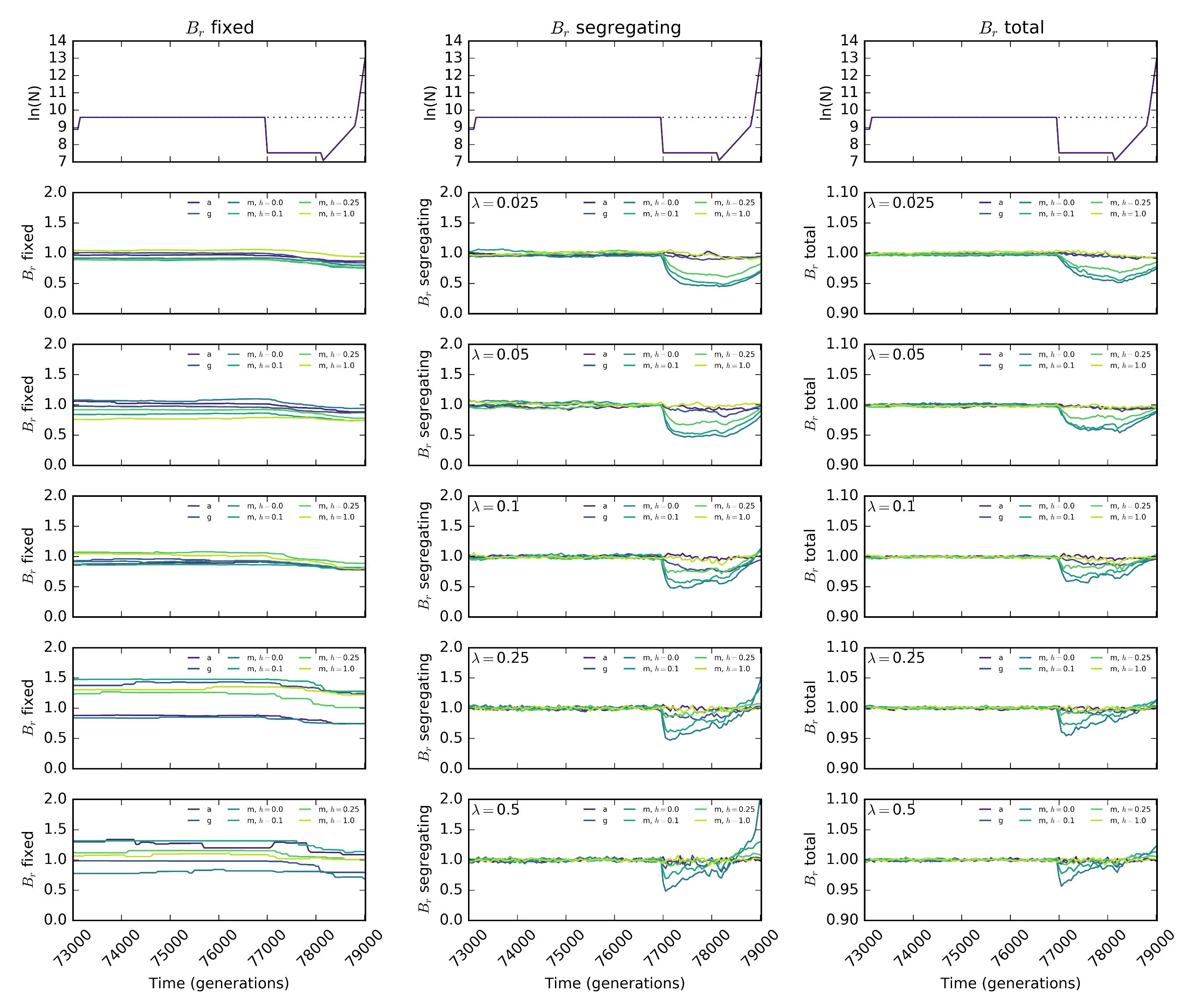

Supplement: S19 Fig — The burden ratio [91] is calculated as the ratio of genetic load between simulations with only ancient growth and those with an additional recent bottleneck and growth. Here load is calculated as the average deviation from optimum fitness due to (left) fixed mutations, (middle) segregating mutations and (right) all mutations. Because of the use of the Gaussian fitness function, the total load is not the sum of the fixed and segregating load. The models shown are: a = additive, g = GBR, and m = multiplicative with varying degrees of dominance (h). For large effect size models, under which there are relatively more mutations that experience strong selection, we see the characteristic drop in the burden ratio following the bottleneck and rebound following re-expansion [91]. (TIFF) [file pgen.1006573.s020.tiff]

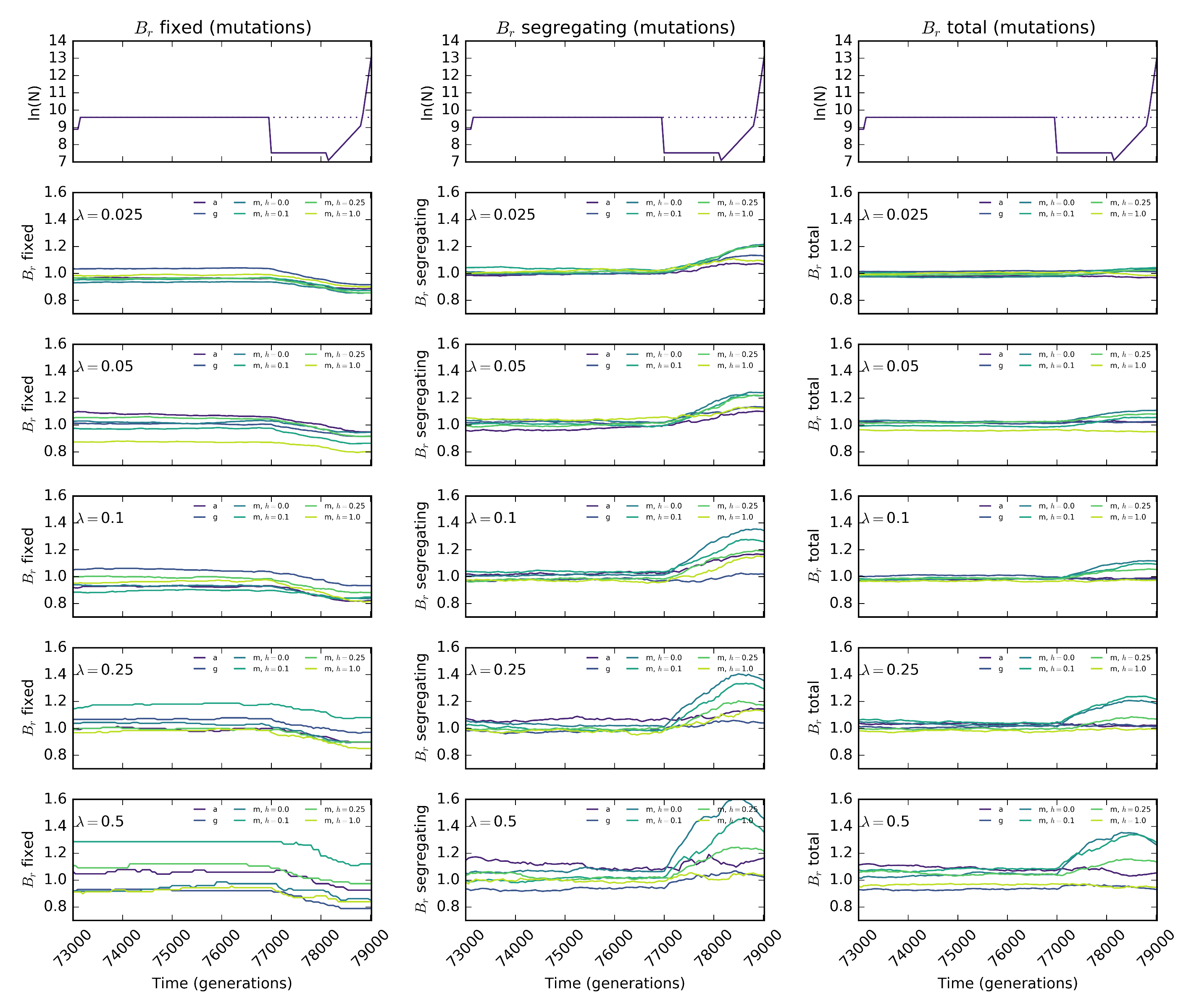

Supplement: S20 Fig — The burden ratio [91] is calculated as the ratio of genetic load between simulations with only ancient growth and those with an additional recent bottleneck and growth. Here load is calculated as the average number of (left) fixed mutations, (middle) segregating mutations and (right) all mutations. The models shown are: a = additive, g = GBR, and m = multiplicative with varying degrees of dominance (h). (TIFF) [file pgen.1006573.s021.tiff]

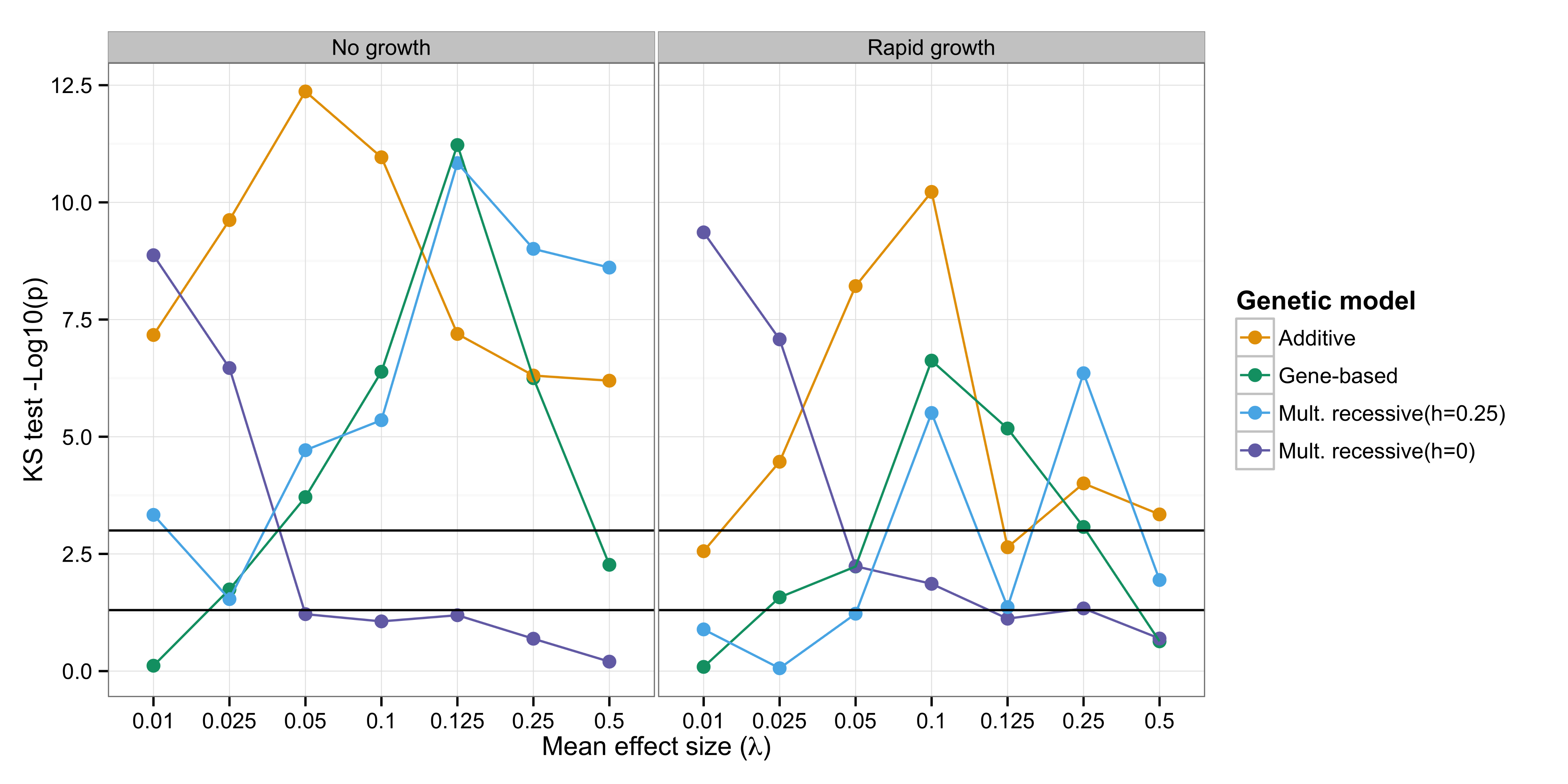

Supplement: S21 Fig — A non-parametric comparison between distribution of allele frequencies between simulated and empirical GWAS hits. Shown are the -log10(p) values from the two-sample Kolmogorov-Smirnov test between the simulated and empirical allele frequencies. The lower and upper horizontal lines show where p = 0.05 and p = 0.001 respectively. Empirical data were downloaded from the NHGRI-EBI GWAS database (http://www.ebi.ac.uk/gwas/) on 02/03/2015, diseases and inclusion criteria are as in [26]. In cases where more than one marker was tied for the lowest p-value, one was chosen at random. Simulated data were subjected to ascertainment sampling such that the MAF distribution of all markers on the simulated genotyping chip was uniform. Specific information regarding the empirical data can be obtained in S1 Table. (TIFF) [file pgen.1006573.s022.tiff]

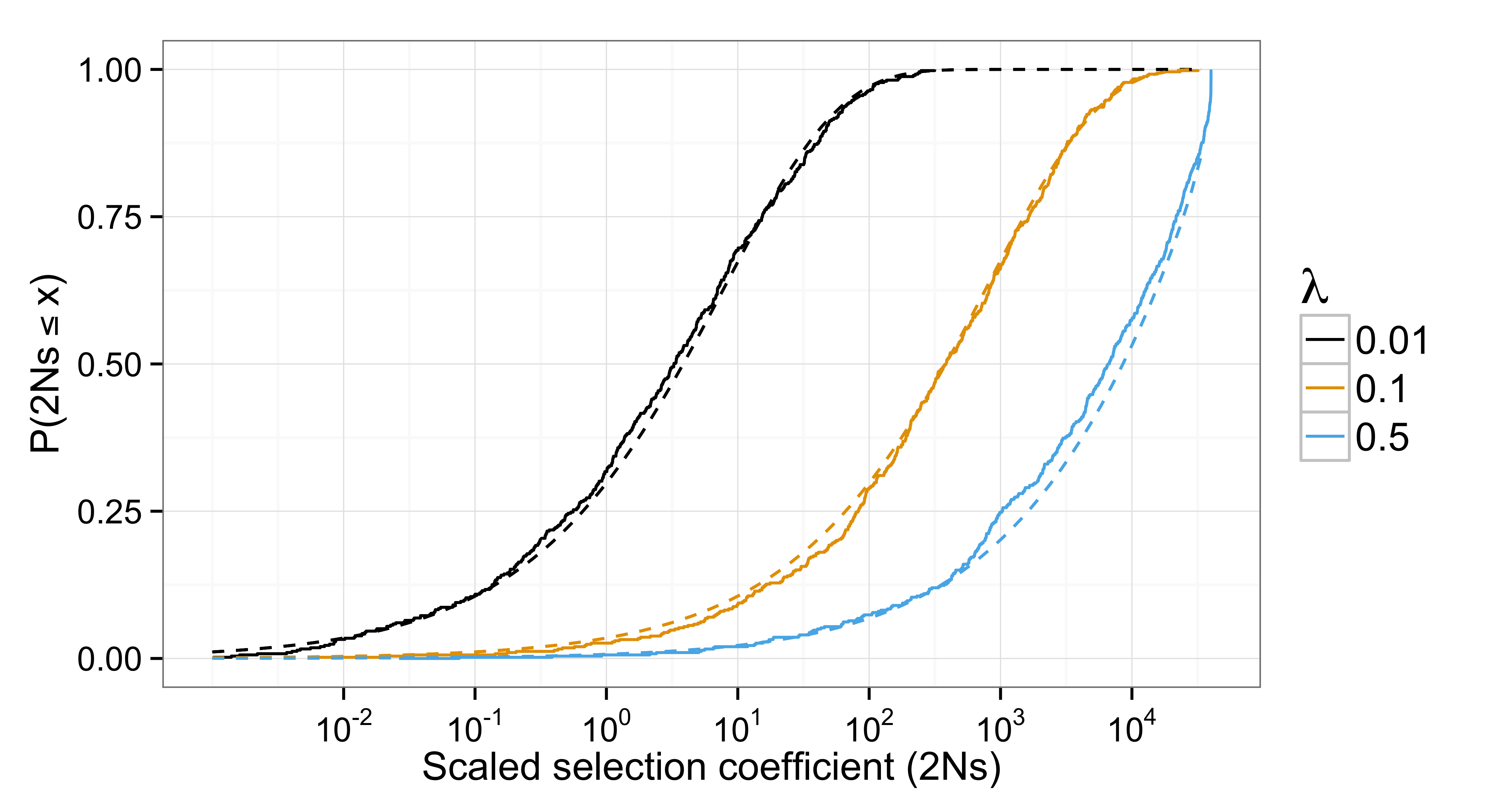

Supplement: S22 Fig — The probability of a new mutation with 2Ns ≤ x on a log scale for various values of λ. The dashed lines show the analytical result and the solid curves are empirical cumulative distribution functions based on a sample of 500 mutation effects from an exponential distribution. The analytical result is an approximation obtained by assuming there is only a single deleterious mutation. (TIFF) [file pgen.1006573.s023.tiff]

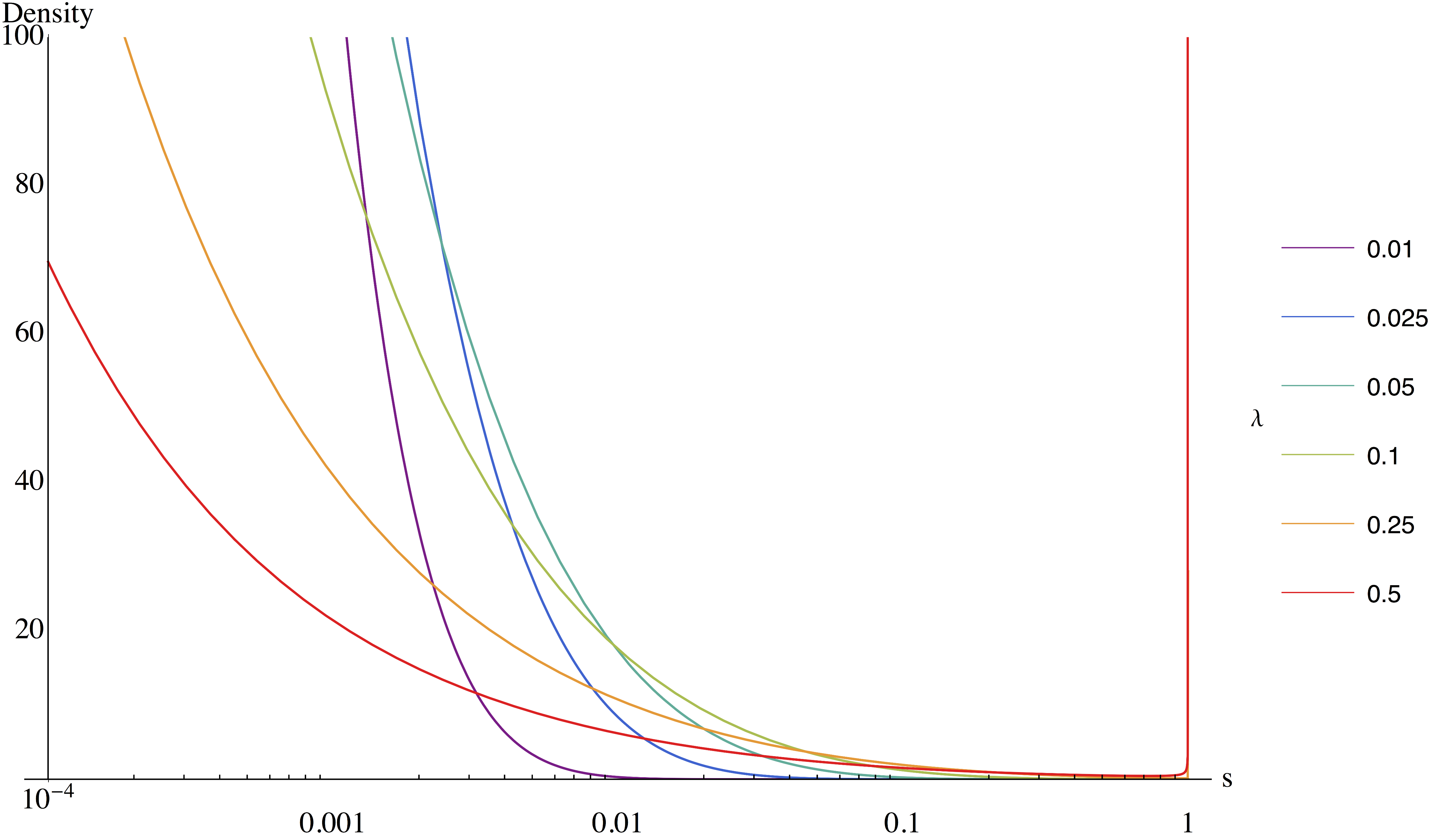

Supplement: S23 Fig — The probability of a new mutation with s = x on a log scale for various values of λ. The analytical result is an approximation obtained by assuming there is only a single deleterious mutation. For λ of 0.25 and 0.5 there is a large mass of lethals near s = 1. (TIFF) [file pgen.1006573.s024.tiff]
